# Modeling and measuring glucose diffusion and consumption by colorectal cancer spheroids in hanging drops using integrated biosensors

**DOI:** 10.1038/s41378-021-00348-w

**Published:** 2022-02-01

**Authors:** Nassim Rousset, Rubén López Sandoval, Mario Matteo Modena, Andreas Hierlemann, Patrick M. Misun

**Affiliations:** ETH Zürich, Department of Biosystems Science and Engineering, Bio Engineering Laboratory, Mattenstrasse 26, CH-4058 Basel, Switzerland

**Keywords:** Electrical and electronic engineering, Physics, Engineering

## Abstract

As 3D in vitro tissue models become more pervasive, their built-in nutrient, metabolite, compound, and waste gradients increase biological relevance at the cost of analysis simplicity. Investigating these gradients and the resulting metabolic heterogeneity requires invasive and time-consuming methods. An alternative is using electrochemical biosensors and measuring concentrations around the tissue model to obtain size-dependent metabolism data. With our hanging-drop-integrated enzymatic glucose biosensors, we conducted current measurements within hanging-drop compartments hosting spheroids formed from the human colorectal carcinoma cell line HCT116. We developed a physics-based mathematical model of analyte consumption and transport, considering (1) diffusion and enzymatic conversion of glucose to form hydrogen peroxide (H_2_O_2_) by the glucose-oxidase-based hydrogel functionalization of our biosensors at the microscale; (2) H_2_O_2_ oxidation at the electrode surface, leading to amperometric H_2_O_2_ readout; (3) glucose diffusion and glucose consumption by cancer cells in a spherical tissue model at the microscale; (4) glucose and H_2_O_2_ transport in our hanging-drop compartments at the macroscale; and (5) solvent evaporation, leading to glucose and H_2_O_2_ upconcentration. Our model relates the measured currents to the glucose concentrations generating the currents. The low limit of detection of our biosensors (0.4 ± 0.1 μM), combined with our current-fitting method, enabled us to reveal glucose dynamics within our system. By measuring glucose dynamics in hanging-drop compartments populated by cancer spheroids of various sizes, we could infer glucose distributions within the spheroid, which will help translate in vitro 3D tissue model results to in vivo.

## Introduction

Three-dimensional (3D) in vitro tissue models, or so-called microtissues, are key in a recent paradigm shift away from 2D biological models for developing new compounds^1^ and testing drug efficacy^2^. There are expectations that these microtissues will replace in vivo models in the long run^1^. 3D microtissues feature more in vivo-like nutrient, metabolite, compound, and waste gradients than their 2D counterparts^3^. However, these gradients lead to heterogeneous metabolism within the 3D in vitro tissue model that is difficult to characterize rigorously for translation to clinical applications^4–6^. Current methods to investigate this heterogeneity include microscopy with fluorescent labels^7^, microtome slicing of fixed tissue models^8^, and fluorescence-assisted flow cytometry of pooled spheroids^9^. These techniques rely on directly probing the inside of the microtissue to determine gradients through its bulk. This can be impractical and time-consuming when determining heterogeneous metabolism and its changes live and in situ.

Another possibility to gain information about microtissue metabolism and consumption characteristics is to use electrochemical biosensors to probe concentrations outside the microtissue. We focus herein on glucose sensing near spheroids, a specific subset of microtissues. Many published or commercialized devices allow live and in situ monitoring of glucose, e.g., by cap sensors that can be integrated into cell culture flasks for adherent or suspension cultures^10^ or by miniaturized sensors integrated into microfluidic systems that enable 2D cell culture^11^ and 3D tissue model^12,13^ measurements^14^. However, to obtain meaningful data to investigate the heterogeneous metabolism of cells in a spheroid, the system must feature a low limit of detection (LoD < 50 μM) to quantify first-order consumption kinetics accurately, a low overall volume to enable rapid metabolism measurements (i.e., fast consumption of available glucose), and stability over several hours to measure concentration changes over time. Additionally, the experimental conditions must be strictly controlled: continuous monitoring is required to determine uptake kinetics at single-spheroid resolution (i.e., no spheroid pooling); volumes need to be known and molecule concentrations in the system need to be controlled (i.e., microenvironments with no dead volumes); and direct microfluidic access to all spheroids is needed to allow a continuous flow at the spheroid surface to prescribe glucose concentrations precisely.

Most of the devices cited above were not suitable for the extraction of metabolic parameters: they work only with larger medium volumes (>50 µL), have a high LoD (>100 µM), are incompatible with spheroids, or require pooling of spheroids. However, there are technologies featuring intricate electrode functionalization methods that enable a sufficiently low detection limit (from 0.3 to 44 μM)^15^. In this work, we made use of the hanging-drop technology^16^ with integrated enzymatic electrochemical glucose biosensors developed in our laboratory^17^. Our biosensors feature simple glucose oxidase enzyme immobilization on electrodes with a glutaraldehyde-based hydrogel. The presence of an air-liquid interface (ALI) in the hanging drop allowed proper tissue oxygenation, fast molecule turnover, and clear spheroid imaging. The integrated biosensors allowed live and in situ glucose monitoring of single spheroids in hanging-drop compartments of less than 7 µL, with a LoD of 0.4 ± 0.1 μM. With direct fluidic access to each monitored spheroid, it was possible to maintain spheroid metabolic activity and to measure metabolic changes over time.

Our integrated hanging-drop biosensors can be used with any scaffold-free 3D biological model^18^. For the proof-of-concept measurements and to test our mathematical model, we used spheroids sourced from a colorectal carcinoma cell line (HCT116). This biological model maintains its spherical structural integrity while resting at the ALI and features well-defined edges, allowing its volume to be measured reliably with a wide-field microscope. Additionally, HCT116 spheroids feature a high rate of aerobic glycolysis, allowing faster metabolism measurements, and are a robust model that enables the use of simplified medium formulations, which further increases control over experimental conditions.

The biosensing principle is based on the enzymatic conversion of glucose by glucose oxidase (GOx), which is immobilized on the integrated electrodes. The enzymatic conversion produces hydrogen peroxide (H_2_O_2_), which is then oxidized at the platinum electrode, yielding a current. The resulting current was measured using an amperometric readout. Operating a device with very low analyte (here glucose) concentrations and low analysis volumes presents particular challenges. At low glucose concentrations, enzymatic conversion by the biosensor and spheroid consumption compete appreciably for glucose. Additionally, the electrode readout—based on H_2_O_2_ oxidation at the electrode surface—is sensitive to H_2_O_2_ and analyte diffusion across the device. Finally, low analysis volumes are susceptible to evaporation, which induces analyte upconcentration. In this work, we present a mathematical model allowing us to account for these phenomena.

For this work, we combined the in situ molecule monitoring capabilities of our hanging-drop-integrated enzymatic glucose biosensors with a physics-based mathematical model of glucose consumption and transport. With this, we investigated glucose diffusion and consumption within and by HCT116 spheroids. We show the analysis pipeline for the specific case of glucose and HCT116 cells in spheroids. This pipeline finds diffusion-consumption parameters specific to the monitored molecule and the studied tissue model. Since glucose is the main carbon source for cellular metabolism, studying the glucose metabolism of cells in a spheroid can be used as a starting point to eventually investigate the metabolism of other molecules, e.g., therapeutic compounds.

## Results

**Fig. 1:**
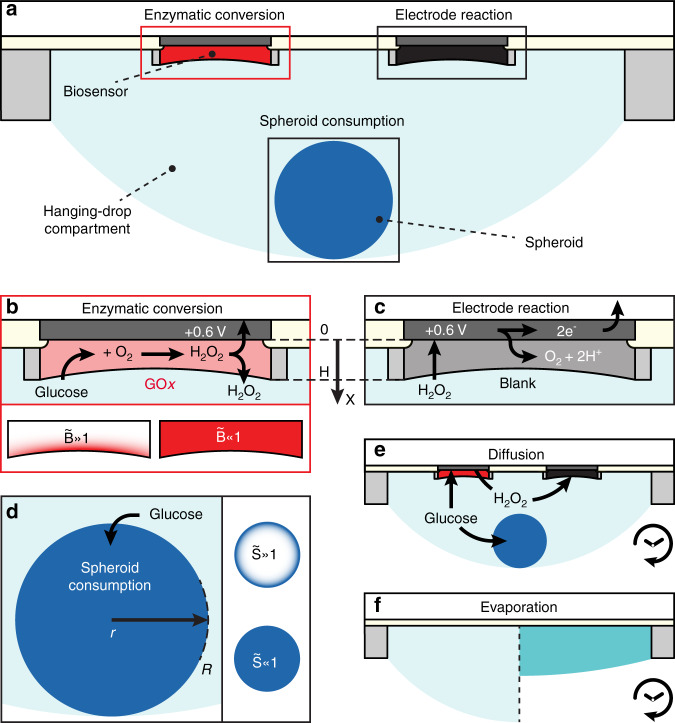
Overview of the processes influencing current measurements. **a** Macroscopic relationship between the glucose-oxidase-functionalized electrode (red), bare-hydrogel-coated platinum electrode (black), and spheroid (blue) all contained within a single hanging-drop compartment allowing single-spheroid-resolution measurements of glucose concentrations. **b** Enzymatic conversion of glucose yielding hydrogen peroxide (H_2_O_2_) by the glucose oxidase (GOx) enzyme modeled with an *x*-axis extending from the platinum electrode surface (x = 0) to the hydrogel-medium interface (x = H). We assume first-order reaction kinetics by the GOx enzyme with a given rate v_g_. The proportion of active enzyme in the functionalized hydrogel is defined by a “biosensor number” $\tilde B$, where a high number implies a consuming surface, and a low number implies a consuming disk. **c** Electrode reaction allowing for amperometric readout of H_2_O_2_ concentrations in the hanging-drop compartment, where the current is proportional to the derivative of H_2_O_2_ concentration at the platinum electrode surface. This electrode reaction also occurs in the GOx-functionalized hydrogel (**b**). **d** Glucose consumption by the spheroid modeled with an *r*-axis defined from the spheroid center (r = 0) to the spheroid-medium interface (r = R). We assume first-order reaction kinetics by the cells in the spheroid with a given rate v_S_. The proportion of active cells in the spheroid is defined by a “spheroid number” $\tilde S$, where a high number implies a consuming shell, and a low number implies a consuming sphere. **e** Over time, diffusion drives the transport of glucose toward the glucose-consuming GOx-functionalized electrode and the glucose-consuming spheroid. It also drives the transport of H_2_O_2_ from the H_2_O_2_-producing GOx-functionalized electrode toward the bare-hydrogel-coated electrode. **f** Evaporation of the hanging drop drives upconcentration of the analytes (glucose and H_2_O_2_), which, in turn, increases the current.

We established the macroscopic relationship between the current, measured by our hanging-drop-integrated enzymatic glucose biosensor, and cellular glucose consumption by a spheroid in the hanging-drop compartment (Fig. 1a). On the microscopic scale, the GOx-functionalized hydrogel of the biosensor oxidizes glucose, yielding H_2_O_2_ (Fig. 1b). The H_2_O_2_ concentration can be readily measured by applying a constant voltage to the integrated electrodes (Fig. 1c). We defined a “biosensor model” linking the biosensor enzymatic conversion of glucose yielding H_2_O_2_ and the subsequent current readout, including glucose and H_2_O_2_ diffusion across the hydrogel. Furthermore, on the microscopic scale, the spheroid consumes glucose (Fig. 1d). We derived a “spheroid model” of the consumption of glucose within spheroids of variable size, including glucose gradients across the spheroids. On the macroscopic scale, glucose and H_2_O_2_ diffuse through the hanging-drop compartment (Fig. 1e), while evaporation causes glucose and H_2_O_2_ upconcentration (Fig. 1f). Throughout the subsequent analysis, we used the results of our precise drop-volume monitoring to account for this upconcentration. By studying the interaction of the aforementioned microscopic components at the macroscopic scale, we were able to precisely estimate the glucose consumption by the spheroid from the current measurements obtained with the integrated electrodes. We defined this overall model as the “measurement model”.

We first introduce the chip and microfluidic designs, then delve into the details of the biosensor, spheroid, and measurement models. We present the fitting strategy required to correctly interpret the measurements obtained by our hanging-drop-integrated enzymatic glucose biosensor. Finally, we use our model to probe the glucose metabolism of cells in 3D spheroids of various sizes.

In the following sections, microscopic-scale concentrations of glucose and H_2_O_2_ will be referred to as c_g_ and c_h_, respectively. Macroscopic-scale bulk concentrations of glucose and H_2_O_2_ near our integrated biosensors will be referred to as $c_g^ \ast$ and $c_h^ \ast$, respectively.

### Hanging-drop-integrated enzymatic glucose biosensor

**Fig. 2:**
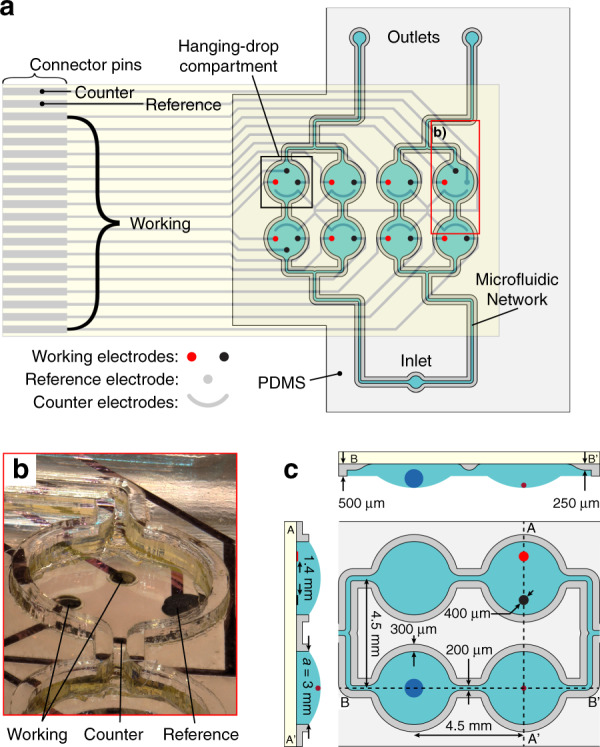
Hanging-drop-integrated glucose biosensor design. **a** Design of the microfluidic channels (PDMS layer in light gray) with rim structures (PDMS layer in dark gray) that confine the medium phase (blue). The flow is driven with a single inlet supplying buffer at ~20 μL min^−1^ and two outlets withdrawing 10 μL min^−1^ each. The inlet and outlets can be interchanged to apply two separate conditions in the same chip. The integrated electrodes on the glass substrate (black and red) are in direct contact with the medium phase and provide single-spheroid resolution in each hanging-drop compartment. The connector pins allow direct connection to a potentiostat. **b** Close-up micrograph of a single hanging-drop compartment within the red box in panel **a** showing the exposed working electrodes, counter electrode, reference electrode, and rim structures. **c** Close-up of the microfluidic channels with various dimensions. Cross-section A–A’ shows the electrodes at the glass-medium interface. Cross-section B–B’ shows a large (blue) and small (red) spheroid at the air-liquid interface. Cross-section B–B’ also shows a fillet geometry transition from the hanging-drop compartment (at 500 μm height) to the channel structures (at 250 μm height) to facilitate medium filling.

The hanging-drop-integrated biosensor comprised 18 channels for electrochemical readouts, which were distributed over eight hanging-drop compartments to enable up to eight simultaneous spheroid measurements. The device also included an on-chip Ag/AgCl reference electrode and platinum counter electrodes in all hanging-drop compartments (Fig. 2a).

The electrodes were connected with a flat-band cable to a custom-made potentiostat integrated into a CMOS chip and a field-programmable gate array module. Both were previously developed by our group^19^. The readout system enabled parallelized current measurements with a noise level lower than 1 pA. Amperometric measurements were carried out using a 3-electrode setup^20^ and by applying a constant potential of +0.65 V to the working electrodes^21^ with regard to the reference electrode (Fig. 2b).

The current and concentration at an electrode in a solution are fundamentally interrelated through the diffusion coefficient of the electroactive species—in our case H_2_O_2_ (D_h_)—, the Faraday constant (F), and the electrode surface area (A)^20^.

1 $$i = FAD_h\left. {\frac{{\partial c_h}}{{\partial x}}} \right|_{x = 0}$$

Our platinum working electrodes featured a 400 μm diameter and were covered with a 5-micron-thick layer of functionalized hydrogel (thickness H). The working principle of the biosensor is presented in Fig. 1b, c. The functionalization consists of a mix of glutaraldehyde, bovine serum albumin, and glucose oxidase (GOx). We optimized the hydrogel functionalization recipe to ensure long-term stability for multiday experiments and deposition repeatability, as evidenced by our ability to reuse the same chip over several days and the tightly spaced calibration curves in Fig. S2d.

Focusing on glucose consumption, the functionalization scheme featured one GOx-functionalized electrode (red electrodes in Fig. 2a) and one or two platinum electrodes coated with hydrogel not containing any GOx, hereafter referred to as “bare-hydrogel-coated” electrodes, (black electrodes in Fig. 2a) per hanging-drop compartment. GOx-functionalized electrodes enabled direct H_2_O_2_ measurement from the enzymatic conversion of glucose, whereas bare-hydrogel-coated electrodes measured diffusive transport timing and accumulation of H_2_O_2_ in the hanging drop. The electrodes were situated at the top of the hanging-drop compartments (Fig. 2c AA’ cross-section). The spheroids rested at the bottom, at the ALI (Fig. 2c BB’ cross-section). Fluidic interconnections between the hanging drops generated two parallel lines with two drops in series (Fig. 2c). The microfluidic network allowed precise control of analyte concentrations, as shown by our numerical simulations of analyte transport (Fig. S1 and Movies S1 and S2).

### Biosensor model

We modeled the working electrodes and hydrogel functionalization at the microscopic scale, which we termed the “biosensor model”. The biosensor model assumed a continuous flow of the analyte of interest with a constant bulk concentration of glucose or H_2_O_2_ ($c_g^ \ast$ or $c_h^ \ast$, respectively) at the biosensor-medium interface (x = H). First, we performed a 1D analysis of H_2_O_2_ (c_h_) diffusion along the *x*-axis (Fig. 1b, c) of our hydrogel. This yielded a relationship between hydrogel thickness and the linear part of the H_2_O_2_ calibration curve. Second, we performed a 1D analysis of glucose (c_g_) and H_2_O_2_ (c_h_) diffusion across the hydrogel layer of GOx-functionalized electrodes. This yielded relationships between the linear part of the glucose calibration curve, H_2_O_2_ and glucose diffusion coefficients (D_h_ and D_g_, respectively), and the enzymatic glucose conversion rate to H_2_O_2_ by GOx (v_g_).

#### Hydrogen peroxide calibration curve

We continuously flowed a buffer with a defined $c_h^ \ast$ through our microfluidic system and waited for a steady-state current level. In this case, H_2_O_2_ transport within the hydrogel layer of both the GOx-functionalized and bare-hydrogel-coated electrodes could be described at the microscale by the following differential equation and boundary conditions:

2 $$\begin{array}{*{20}{c}} {D_h\frac{{\partial ^2c_h}}{{\partial x^2}} = 0,} & {c_{h\left( {x = 0} \right)} = 0,} & {c_{h\left( {x = H} \right)} = c_h^ \ast } \end{array}$$

By solving the set of equations in Eq. (2) with the current relationship at the electrode from Eq. (1), we found the concentration dependence of the current:

3 $$i\left( {c_h^ \ast } \right) = \underbrace {FA\frac{{D_h}}{H}}_{H_2O_2\;{{{\mathrm{calibration}}}}\;{{{\mathrm{slope}}}}}c_h^ \ast$$

H denotes the hydrogel layer thickness (Fig. 1b). The H_2_O_2_ diffusion coefficient (D_h_) in a glutaraldehyde-based hydrogel is 0.25–0.40 times that in sodium phosphate buffer^22^. As H can vary slightly between experimental runs, the ratio D_h_/H can be determined with H_2_O_2_ flow calibration. Such calibration could allow us to measure hydrogel functionalization thickness to account for less precise deposition techniques.

After stopping the buffer with a defined $c_h^ \ast$ of 1 μM (Fig. S4b), the H_2_O_2_ concentration gradually decreases over several hours. This shows that H_2_O_2_ is actively broken down by the electrodes.

#### Glucose calibration curve

The GOx functionalization on the electrodes oxidizes glucose, yielding H_2_O_2_ in a one-to-one reaction (Fig. 1b schematic) at a given first-order rate ($v_g$). We continuously flowed a buffer with a defined $c_g^ \ast$ and waited for a steady-state current level. Continuous flow ensured that the produced H_2_O_2_ would be readily washed away from the electrode and would not accumulate in the hanging-drop compartments. This steady-state condition can be described, at the microscale, by the following differential equations and boundary conditions for glucose (4a) and H_2_O_2_ (4b):

4a $$\begin{array}{*{20}{c}} {D_g\frac{{\partial ^2c_g}}{{\partial x^2}} - v_gc_g = 0,} & {\left. {\frac{{\partial c_g}}{{\partial x}}} \right|_{x = 0} = 0,} & {c_{g\left( {x = H} \right)} = c_g^ \ast } \end{array}$$

4b $$\begin{array}{*{20}{c}} {D_h\frac{{\partial ^2c_h}}{{\partial x^2}} + v_gc_g = 0,} & {c_{h\left( {x = 0} \right)} = 0,} & {c_{h\left( {x = H} \right)} = 0} \end{array}$$

Finding c_g_(x) (Supplementary Information S1) with the glucose equations (4a) yielded the dimensionless “biosensor number” $\tilde B = H\sqrt {v_g/D_g}$, which features two extreme cases: $\tilde B \ll 1$ with a consuming hydrogel surface and $\tilde B \gg 1$ with a consuming hydrogel bulk (Fig. 1b). The glucose diffusion coefficient (D_g_) in a glutaraldehyde-based hydrogel is 0.062–0.086 times that in sodium phosphate buffer^22^. By integrating c_g_(x) times v_g_ over the hydrogel volume and dividing the result by the hydrogel volume, we found the average first-order consumption of glucose by the GOx enzyme (Q_E_):

5 $$Q_E = \frac{{A\mathop {\int }\nolimits_0^H c_g\left( x \right)v_gdx}}{{AH}} = \underbrace {\frac{{\sqrt {v_gD_g} }}{H}\tanh \left( {\tilde B} \right)}_{q_E}c_g^ \ast$$

The relationship between a bulk concentration of glucose $c_g^ \ast$ and its consumption by the enzymes in the hydrogel is, therefore, of the first order: $Q_E = q_Ec_g^ \ast$. We then replaced c_g_(x) in the H_2_O_2_ differential equations (4b) to find c_h_(x) (Supplementary Information S1). By using the current-diffusive-flux relationship at the electrode from Eq. (1), the first derivative of c_h_(x) at the electrode surface (x = 0) yielded the concentration dependence of the current:

6 $$i( {c_g^ \ast } ) = \underbrace {FA\frac{{D_g}}{H}\left( {1 - {{{\mathrm{sech}}}}\left( {\tilde B} \right)} \right)}_{{{{\mathrm{Glucose}}}}\;{{{\mathrm{calibration}}}}\;{{{\mathrm{slope,}}}}\;b}c_g^ \ast$$

**Fig. 3:**
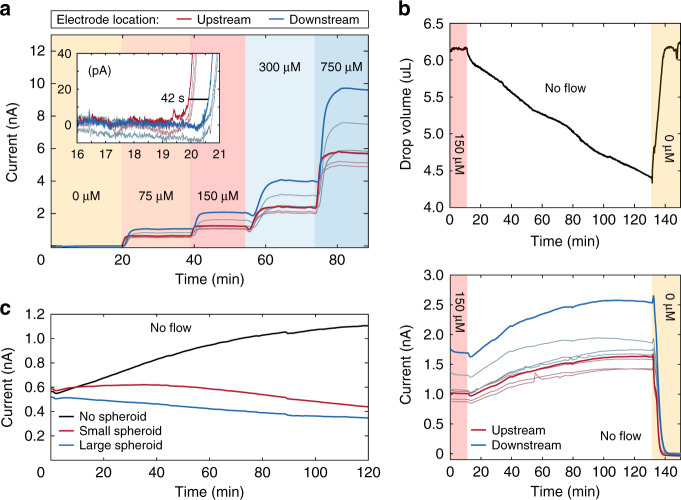
Experimental data associated with elements of the measurement model. The working electrodes have a diameter of 0.4 mm; therefore, currents of 1 nA correspond to a current density of 2 nA mm^−^^2^. **a** Enzymatic conversion of glucose to generate a current readout: We show the current response of all 8 glucose-oxidase-functionalized electrodes and highlight the response of an upstream (red) and downstream (blue) electrode in a single line. We plot the signal over time of a typical 3-point flow calibration curve from 0 to 150 μM, followed by an investigation of the linear range from 300 to 750 μM. The inset of this figure shows raw data at the first step from 0 to 75 μM. The inset also shows a clear separation between upstream and downstream electrode responses, as the glucose reaches the upstream electrodes 42 seconds before it reaches the downstream electrodes. This inset also shows the data used to calculate the limit of detection of 0.4 ± 0.1 μM. **b** Evaporation: The top plot shows the experimental drop-volume measurement, which allows us to fit a linear evaporation rate. The target volume is equivalent to a hanging-drop height of 600 μm. The experimental protocol involves flushing a PBS buffer, spiked with 150 μM glucose, stopping the flow (no flow), and then flushing with PBS buffer containing 0 μM glucose. The bottom plot shows the current response over time, directly following the response of panel **a**, of a typical hydrogel characterization in the absence of spheroids. The current increases due to a combination of evaporation/upconcentration and H_2_O_2_ accumulation in the hanging-drop compartment. **c** Spheroid metabolic activity: Comparison of metabolism measurements without a spheroid (black), a small spheroid (red), and a large spheroid (blue). The current measured near large spheroids drops faster than that measured near small spheroids. The combination of all the effects described in Fig. 1 gives rise to the differences in current observed here. This is why an analytical model is necessary.

Therefore, with a simple flow calibration scheme to find the glucose calibration slope (b) as in Fig. 3a, we could determine the biosensor number $\left( {\tilde B} \right)$. The relationship between b in equation (6) (Fig. S2) and the average consumption first-order constant q_E_ in Eq. (5) is given by the following equation:

7 $$\frac{{q_E}}{b} = \frac{{\frac{{\sqrt {v_gD_g} }}{H}\tanh \left( {\tilde B} \right)}}{{FA\frac{{D_g}}{H}\left( {1 - {{{\mathrm{sech}}}}\left( {\tilde B} \right)} \right)}} = \frac{{\tilde B}}{{FAH}}\coth \left( {\frac{{\tilde B}}{2}} \right)$$

Typical experimental values of the sensitivity (b), q_E_ and $\tilde B$, and the stability of the biosensor over multiple measurements are shown in Fig. S8. This figure shows that the biosensor sensitivity changes over the course of multiple measurements: going from 9 ± 2 nA mM^−1^ (or 18 ± 4 nA mM^−1^ mm^−2^) down to 3.1 ± 0.7 nA mM^−1^ (or 6.2 ± 1.4 nA mM^−1^ mm^−2^) over 10 successive measurements. In situ flow calibrations were used to determine this sensitivity change during the experimental workflow (Fig. 3a). Figure 3a inset also shows low noise in the raw signal, leading to our glucose LoD of 0.4 ± 0.1 μM calculated with a parametric method^23^ and using the data from all eight GOx-functionalized electrodes. The LoD of H_2_O_2_ is also relevant because the measurement is made by amperometric readout through oxidation of H_2_O_2_. We found that a H_2_O_2_ concentration of as little as 0.7 μM results in a discernable step increase in the current for all hydrogel-coated electrodes (Fig. S4b inset). Finally, Fig. S2d shows a glucose-sensor linear range up to 750 μM, where the deviation from linearity is ~6%.

### Spheroid model

We performed a similar microscopic-scale analysis to that for the biosensor model to model glucose transport and consumption in the spheroid, which we termed the “spheroid model”. We started by solving the diffusion-reaction equation with first-order consumption kinetics (v_S_) in a spherical geometry as defined in Fig. 1d. By continuously flowing a solution with a defined bulk glucose concentration ($c_g^ \ast$), the steady-state solution of glucose concentration in the spheroid c_g_(r) is described at the microscale by the following differential equations and boundary conditions:

8 $$\begin{array}{*{20}{c}}\displaystyle {D_S\frac{1}{{r^2}}\frac{\partial }{{\partial r}}\left( {r^2\frac{\partial }{{\partial r}}c_g\left( r \right)} \right) - v_Sc_g\left( r \right) = 0,} & {\left. \displaystyle {\frac{{\partial c_g}}{{\partial r}}} \right|_{r = 0} = 0,} & {c_{g\left( {r = R} \right)} = c_g^ \ast } \end{array}$$

Finding c_g_(x) (Supplementary Information S1) yields the dimensionless “spheroid number” $\tilde S = R\sqrt {v_S/D_S}$, which features two extreme cases: $\tilde S \ll 1$ with a consuming sphere and $\tilde S \gg 1$ with a consuming shell (Fig. 1d). The spheroid radius (R) was measured by wide-field microscopy imaging. The v_S_ constant of glucose by the cells and the diffusion coefficient of glucose through the spheroid (D_S_) are unknown. By integrating c_g_(r) times v_S_ over the spheroid volume dV and dividing the result by the spheroid volume, we found the average consumption of glucose by the spheroid (Q_S_).

9 $$Q_S = \frac{{{\int} {c_g\left( r \right)v_SdV} }}{{4\pi R^3/3}} = \underbrace {3\frac{{D_S}}{{R^2}}\left( {\tilde S\coth \tilde S - 1} \right)}_{q_S}c_g^ \ast$$

The relationship between the bulk concentration of glucose $c_g^ \ast$ and its consumption by the spheroid is therefore $Q_S = q_Sc_g^ \ast$.

### Measurement model

It is necessary to stop the flow for a metabolism measurement to generate a measurable variation in $c_g^ \ast$ in the hanging-drop compartment through spheroid consumption of glucose. An example of a biosensor measurement in an empty hanging drop without flow is reported in Fig. 3b. Figure 3c shows a metabolism measurement with an empty hanging drop, one populated by a small spheroid, and one populated by a large spheroid. Interpreting this figure required the definition of a “measurement model”.

As shown in Fig. 1, the following phenomena contributed to the measured current in the absence of flow: (i) enzymatic conversion of glucose and production of H_2_O_2_, (ii) Faradaic electrode oxidation of H_2_O_2_, (iii) spheroid consumption of glucose, (iv) glucose and H_2_O_2_ diffusion, and (v) medium evaporation. We performed an analysis to decouple the listed phenomena affecting current readout and to accurately determine the bulk glucose concentration in the hanging-drop compartment.

Here, we briefly elaborate on the aforementioned phenomena. (i) Enzymatic conversion: Eq. (5) from the biosensor model outlines that GOx functionalization consumes glucose and produces H_2_O_2_ at a rate $Q_E = q_Ec_g^ \ast$ [mol m^−3^ s^−1^] (Fig. 1b). (ii) Electrode activity: The oxidation of H_2_O_2_ at the platinum electrode surface (Fig. 1c) drives a diffusive H_2_O_2_ flux in the hanging-drop compartment toward the electrode that is directly proportional to its bulk concentration $c_h^ \ast$. This is effectively H_2_O_2_ consumption at a rate $Q_e = q_ec_h^ \ast$ [mol m^−3^ s^−1^] (Fig. S4). (iii) Spheroid activity: Our goal is to measure glucose consumption q_S_ by the spheroid (Fig. 1d). Glucose consumption by the spheroid competes with enzymatic glucose conversion by the biosensor, reducing H_2_O_2_ production. Equation (9) from the spheroid model indicates that the spheroid consumes glucose at a rate $Q_S = q_Sc_g^ \ast$ [mol m^–3^ s^−1^]. (iv) Diffusion effects: As H_2_O_2_ is produced, it diffuses to and from neighboring electrodes (Fig. 1e). In the measurement model, this diffusion is neglected. We elaborate on the preponderance of diffusion in the next section. (v) Evaporation effects: Medium evaporation causes analyte upconcentration in the hanging drop (Fig. 1f), which causes an upward current drift. Assuming a constant evaporation rate (V_t_) and an initial hanging-drop compartment volume (V_0_), Eq. (10) defines the volume change over time.

10 $$\begin{array}{*{20}{c}} {\frac{{\partial V}}{{\partial t}} = - V_t,} & {V\left( t \right) = V_0 - V_tt} \end{array}$$

Our setup enabled continuous drop-height monitoring, which readily returned the experimental value of V(t) over the course of a measurement (Fig. 3b). We expanded the total derivative of concentration, given by the number of molecules n(t) per unit volume c(t) = n(t)/V(t), to find the upconcentration term per Eq. (11). This evaporation-correction term is necessary to define the set of differential equations describing the rate of change of the bulk concentrations ($c_g^ \ast$ and $c_h^ \ast$).

11 $$\frac{{dc}}{{dt}} = \frac{{\partial c}}{{\partial n}}\frac{{\partial n}}{{\partial t}} + \frac{{\partial c}}{{\partial V}}\frac{{\partial V}}{{\partial t}} = \frac{{\partial c}}{{\partial t}}\underbrace { - \frac{c}{V}\frac{{\partial V}}{{\partial t}}}_{{{{\mathrm{Upconcentration}}}}\;{{{\mathrm{term}}}}}$$

#### Current readout

In contrast to the previous biosensor and spheroid models, the measurement model does not consider perfused experimental conditions. The measurement model applies to the “no flow” results shown in Fig. 3b and c. Therefore, the current described by Eq. (1) takes a different form. The steady-state diffusion layer model^20^ establishes that the current is directly proportional to the H_2_O_2_ concentration in the bulk $c_h^ \ast$. The mass transport coefficient (m_h_) is the constant determining this proportionality.

12 $$i\left( t \right) = FAm_hc_h^ \ast \left( t \right)$$

#### Biosensor measurement model solution

Enzyme, electrode, and spheroid activity—combined with Eqs. (10) and (11) to account for upconcentration—define a set of differential equations. Differential Eqs. (13) and (14) define the rate of change of $c_g^ \ast \left( t \right)$ and $c_h^ \ast \left( t \right)$, respectively. The initial conditions are set precisely as medium flow is stopped, such that $c_g^ \ast \left( {t = 0} \right) = c_{g0}^ \ast$ and $c_h^ \ast \left( {t = 0} \right) = c_{h0}^ \ast$, where $c_{g0}^ \ast$ is the experimentally prescribed glucose concentration of 150 μM.

13 $$\frac{{dc_g^ \ast }}{{dt}} = - q_Ec_g^ \ast - q_Sc_g^ \ast + \frac{{c_g^ \ast }}{{V\left( t \right)}}V_t$$

14 $$\frac{{dc_h^ \ast }}{{dt}} = q_Ec_g^ \ast - q_ec_h^ \ast + \frac{{c_h^ \ast }}{{V\left( t \right)}}V_t$$

Differential Eq. (13) can be solved directly to find $c_g^ \ast \left( t \right)$ because it does not depend on $c_h^ \ast \left( t \right)$.

15 $$c_g^ \ast \left( t \right) = \frac{{c_{g0}^ \ast }}{{1 - \frac{{V_t}}{{V_0}}t}}e^{ - \left( {q_E + q_S} \right)t}$$

Using $c_g^ \ast \left( t \right)$ defined in Eq. (15) to solve Eq. (14) yields an analytical expression of $c_h^ \ast \left( t \right)$:

16 $$c_h^ \ast \left( t \right) = \frac{1}{{1 - \frac{{V_t}}{{V_0}}t}}\left[ {c_{h0}^ \ast e^{ - q_et} + c_{g0}^ \ast \frac{1}{{\left( {1 + \frac{{q_S - q_e}}{{q_E}}} \right)}}\left( {e^{ - q_et} - e^{ - \left( {q_E + q_S} \right)t}} \right)} \right]$$

Inserting $c_h^ \ast \left( t \right)$ into Eq. (12) gives the analytical expression of the measured current over time.

17 $$i\left( t \right) = \frac{{FAm_h}}{{1 - \frac{{V_t}}{{V_0}}t}}\left[ {c_{h0}^ \ast e^{ - q_et} + c_{g0}^ \ast \frac{1}{{\left( {1 + \frac{{q_S - q_e}}{{q_E}}} \right)}}\left( {e^{ - q_et} - e^{ - \left( {q_E + q_S} \right)t}} \right)} \right]$$

#### List of parameters

The current solution depends on a plethora of parameters. However, most of these are known or experimentally determined constants.

The following parameters are known in Eq. (17). Units were chosen to give nA for currents and mol μL^−1^ for concentrations.

- F: Faraday constant, or the amount of charge per mole of electrons (nC mol^−1^);
- A: Electrode surface area (mm^2^)—determined by the design;
- V_t_: Hanging-drop evaporation rate (mm³ min^−1^)—determined by monitoring drop height;
- V_0_: Initial volume in the hanging drop (mm³ = μL)—determined by monitoring drop height;
- $c_{g0}^ \ast$: Experimental initial bulk glucose concentration in the system (mol mm^−3^);
- q_E_: Enzymatic glucose conversion rate (min^−1^)—known from calibration curves and Eq. (5).

The following parameters are unknown.

- q_e_: Electrode reaction rate of hydrogen peroxide (min^−1^)—unknown, constant;
- $c_{h0}^ \ast$: Initial hydrogen peroxide concentration in the system (mol mm^−3^)—unknown, variable;
- m_h_: Mass transport coefficient of hydrogen peroxide (mm s^−1^)—unknown, constant;
- q_S_: Spheroid consumption of glucose (min^−1^)—unknown, variable.

To find the various parameters, we performed two consecutive sets of measurements: a calibration measurement to determine electrode and enzyme characteristics, followed by a metabolism measurement to determine spheroid consumption.

### Diffusion preponderance

**Fig. 4:**
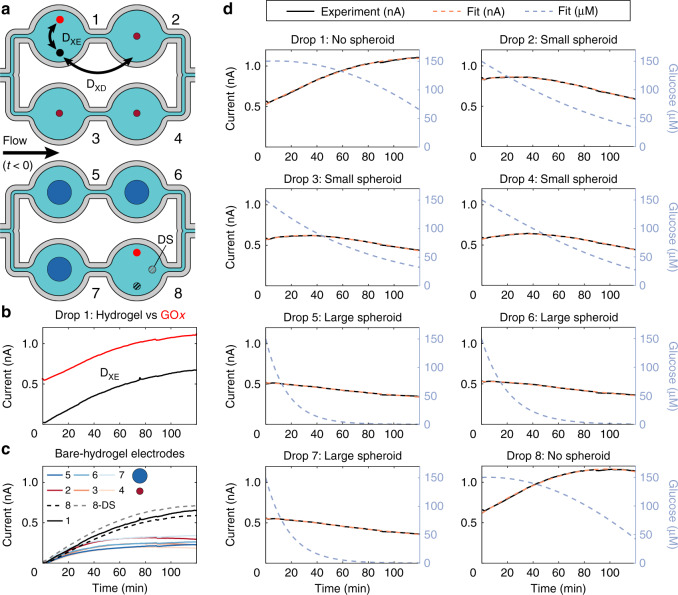
Experimental data and model fit. The working electrodes have a diameter of 0.4 mm; therefore, currents of 1 nA correspond to a current density of 2 nA mm^−2^. **a** Experimental layout with the numbering of hanging-drop compartments and what populates them—either a large spheroid (blue sphere), a small spheroid (red sphere), or no spheroid. A schematic of cross-electrode diffusion (D_XE_) and cross-drop diffusion (D_XD_) shows the possibility of glucose and H_2_O_2_ transport through the system and how our bare-hydrogel-coated electrodes can quantify this transport. An arrow describing the direction of flow prior to the start of the measurements allows us to define upstream (drops 1, 3, 5, and 7) and downstream (2, 4, 6, and 8) drops. Hashed bare-hydrogel-coated electrodes are shown in drop 8 to highlight the downstream bare-hydrogel-coated electrode (DS) in gray, which will be plotted as a gray dashed line (8-DS) in subfigure **c**. **b** Comparison between the bare-hydrogel-coated electrode signal (black line) and the GOx-functionalized signal (red line) in Drop 1, a control drop with no spheroid. Since the bare-hydrogel-coated electrode measures the H_2_O_2_ concentration around it but does not generate H_2_O_2_, this figure shows that we can directly measure cross-electrode diffusion D_XE_ from the GOx-functionalized electrode. **c** Current signal on all bare-hydrogel-coated electrodes through all drops, demonstrating a similar response across spheroid-populated drops and a distinctly different response in the empty drops. The current measured at bare-hydrogel-coated electrodes is characteristic of bulk H_2_O_2_ concentration in the hanging-drop compartment. As shown in Fig. S4c, a current of 0.2–0.5 nA results from a bulk H_2_O_2_ concentration between 2 and 7 µM. In the case of drop 8, where two bare-hydrogel-coated electrodes are present, the electrode closest to the GOx-functionalized electrode (dashed gray line 8-DS) shows a higher current than the furthest electrode (dashed black line). This shows the D_XE_ effect. **d** Experimental current data (continuous black line) and fitted sum of exponential currents (orange dotted lines) for the GOx-functionalized electrodes. The sum of exponentials provides an excellent fit for the current data. Spheroid sizes and fitted proportional coefficients and exponential coefficients, used to plot the current fits, are enumerated in Table 1. The glucose concentration dynamics giving rise to such a current fit are shown (blue dotted line).

We defined two main diffusion length scales in Fig. 4a: cross-electrode diffusion (D_XE_) between electrodes in a hanging-drop compartment and cross-drop diffusion (D_XD_) between electrodes in neighboring hanging-drop compartments. We quantified the preponderance of diffusion across the microfluidic network using a special electrode functionalization scheme presented in Fig. S3. We showed that our experimental functionalization scheme—enabling simultaneous GOx-functionalized and bare-hydrogel-coated electrode measurements in each hanging-drop compartment per Fig. 2a—allowed us to neglect D_XD_ and quantify D_XE_ (Supplementary Information S2). In short experiments, D_XD_ can be safely neglected between drops with similar conditions (i.e., drops hosting a spheroid of a defined size). In all experiments, our bare-hydrogel-coated electrodes neighboring our GOx-functionalized electrodes quantified D_XE_ (Fig. 4b). Our bare-hydrogel-coated electrodes additionally provided a direct quantification of H_2_O_2_ accumulation in each hanging-drop compartment (Fig. 4c), which enabled us to demonstrate the competitive glucose consumption between GOx enzymes and the spheroid.

### Current data fitting

Unravelling the current response required analysis of the raw data of the potentiostatic readout. We applied a low-pass filter to the raw data shown in the inset of Fig. 3a (raw vs. filtered measurement data in Fig. S5). Additionally, by monitoring the drop volume over time, we found that the evaporation rate was comparably constant during a measurement cycle (Fig. 3b). We applied a linear evaporation-correction term by multiplying the current i(t) by the denominator of Eq. (17). The evaporation-corrected current (y(t)) allows us to separate the consumption kinetics from the evaporation kinetics. Further factoring the exponential terms in the result of Eq. (17) gives the following:

18 $$y\left( t \right) = i\left( t \right)\left( {1 - \frac{{V_t}}{{V_0}}t} \right) = FAm_h\left[ {\left( {c_{h0}^ \ast + \frac{{c_{g0}^ \ast }}{{1 + \frac{{q_S - q_e}}{{q_E}}}}} \right)e^{ - q_et} - \frac{{c_{g0}^ \ast }}{{1 + \frac{{q_S - q_e}}{{q_E}}}}e^{ - \left( {q_E + q_S} \right)t}} \right]$$

Ultimately, Eq. (18) highlights that the concentration and current dynamics in our hanging-drop-integrated biosensor can be described by a sum of two exponentials. In our model, the parameters should be defined as follows:

19 $$y \left( t \right) = a \exp \left( {bt} \right) + c \exp \left( {dt} \right), \,{\text{where}}\quad \left\{ \begin{array}{*{20}{l}} {a = FAm_h\left[ {c_{h0}^ \ast + c_{g0}^ \ast \frac{{q_E}}{{q_E + q_S - q_e}}} \right]} \hfill \\ {b = - q_e} \hfill \\ {c = - FAm_hc_{g0}^ \ast \frac{{q_E}}{{q_E + q_S - q_e}}} \hfill \\ {d = - \left( {q_E + q_S} \right)} \hfill \end{array} \right.$$

Fitting these two exponentials using two pseudoinverses of the experimental data (Supplementary Information S3) allows finding all parameters without having to choose initial parameter guesses. This fit was done to find the parameters in Table 1, which were used to plot the fitted curves in Fig. 4d. The coincidence between the experimental current (full black line in Fig. 4d) and the fitted current (dashed orange line in Fig. 4d) provided evidence that the current dynamics can be completely explained by a sum of two exponentials. This result gives us confidence that our model correctly accounts for the different phenomena affecting the glucose-current relationship and enables us to estimate the actual glucose concentration (dashed blue line in Fig. 4d) near the biosensing electrode in the hanging drop. There are various special cases for the values of the proportional coefficients a and c and of the exponential coefficients b and d that we elaborate on in the Supplementary Information S4.

**Table 1:** List of fitting parameter solutions for Fig. 4d fitting data results with the real and imaginary parts of the proportional coefficients a and c and the exponential coefficients b and d.

			a	a	b	b	c	c	d	d
Drop	Electrode	Spheroid	Real	Imag	Real	Imag	Real	Imag	Real	Imag
#	#	μm	nA	nA	min^−1^	min^−1^	nA	nA	min^−1^	min^−1^
1	**2**	None	0.2617	0.4076	−0.0015	−0.0099	0.2617	−0.4076	−0.0015	0.0099
2	**17**	285	0.4145	0.7545	−0.0115	−0.0073	0.4145	−0.7545	−0.0115	0.0073
3	**4**	286	0.2841	1.2274	−0.0142	−0.0044	0.2841	−1.2274	−0.0142	0.0044
4	**15**	286	0.2863	0.5251	−0.0110	−0.0088	0.2863	−0.5251	−0.0110	0.0088
5	**6**	452	0.5481	0	−0.0066	0	−0.0339	0	−0.0618	0
6	**13**	453	0.6037	0	−0.0070	0	−0.0762	0	−0.0497	0
7	**9**	453	0.6107	0	−0.0071	0	−0.0608	0	−0.0577	0
8	**10**	None	0.3083	0.4383	−0.0016	−0.0110	0.3083	−0.4383	−0.0016	0.0110

### Advection and diffusion within hanging drops

We used the finite element method to numerically model advective and diffusive transport within our hanging drops (Fig. S1). We used the parameters determined by fitting our measurement model to the experimental data (Table 1) to solve the numerical models.

Figure S1a shows that the normalized glucose concentration at the bare-hydrogel-coated electrode labeled D_XE_ (Fig. 4a) is 1 during flow. However, Fig. S1b shows a 1% decrease in glucose concentration for the second drop in series, labeled “Downstream blanks”. The nonzero H_2_O_2_ concentration in downstream bare-hydrogel-coated electrodes further shows the effect of hanging-drop compartment serialization (Fig. S1c). The results from our model (Fig. S1) and the experimental diffusion results (Fig. S3) underline that drop serialization should be kept at a minimum when working with low analyte concentrations.

Additionally, the numerical model shows that the bulk H_2_O_2_ concentration in a hanging-drop compartment with a spheroid is ~2 μM (Fig. S1c). We validated this estimation by including a H_2_O_2_ calibration curve (Fig. S4b), which indicates that the maximal currents measured with bare-hydrogel-coated electrodes (Fig. 4c), in the 0.2–0.5 nA range, result from a bulk H_2_O_2_ concentration between 2 and 7 μM.

### Spheroid-size dependence of fitted consumption parameters

Using the result from our spheroid model and Eq. (9), we find the relation between the experimental values of q_S_ and *R* and the spheroid number $\tilde S$.

20 $$q_S = 3\frac{{D_S}}{{R^2}}\left( {\tilde S\coth \tilde S - 1} \right) \to \frac{{q_SR^2}}{{3D_S}} = \tilde S\coth \tilde S - 1$$

**Fig. 5:**
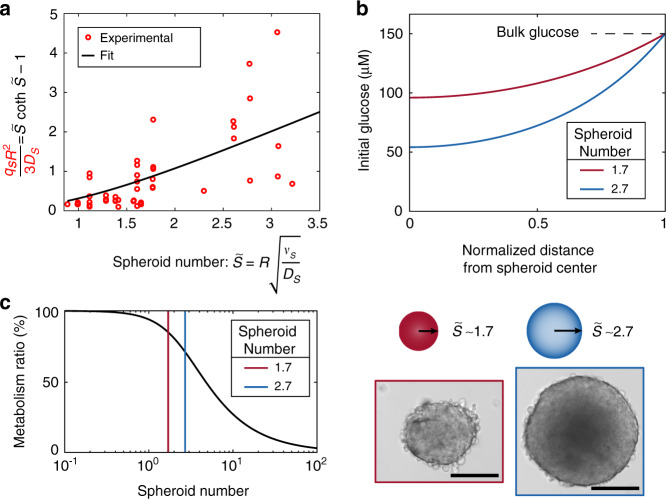
Spheroid number and its implications. **a** The spheroid number fit uses the known data points found by using the experimental values of q_S_, R, and D_S_ with the part of the equation in red (*y*-axis) to fit the value of $\tilde S$ with the part of the equation in black (*y*-axis). **b** Two spheroids were chosen as typical. The small spheroid (red) with a 1.7 spheroid number has a diameter of 285 μm, and the large spheroid (blue) with a 2.7 spheroid number has a diameter of 453 μm. The scale bar is 200 μm. The images were taken at the initial time point, when the bulk glucose levels were $c_{g0}^ \ast$ = 150 μM in the hanging-drop compartment. With this initial glucose level, we plotted the expected glucose distribution through the spheroids for the spheroid numbers fitted in subfigure **a**. The radial distance from the center of the microtissue was normalized by the spheroid radius to emphasize the difference in the expected initial glucose distribution. The heterogeneous glucose distribution will lead to a metabolism that diverges from ideal homogeneous consumption. **c** The proportion of cells that participate in the metabolization of glucose (metabolism ratio) is plotted as a function of the spheroid number. This function is found by normalizing Eq. (9) by an assumption that all cells in the spheroid consume glucose uniformly: $\frac{{Q_S}}{{c_g^ \ast v_S}} = \frac{3}{{\tilde S^2}}\left( {\tilde S\coth \tilde S - 1} \right)$. At low spheroid numbers, 100% of cells participate in metabolic activity; at high spheroid numbers, only a few cells participate in metabolic activity. The relative metabolic activities of the typical spheroids are 85% and 70% for the smaller and larger spheroids, respectively.

The left side of the resulting Eq. (20) was experimentally determined and plotted in Fig. 5a. The experimentally measured radii (R) of HCT116 spheroids, formed by initial cell seeding of 150 and 900 cells, are shown in Fig. S6a. As an endpoint characterization of spheroid viability, we performed size-dependent measurements of spheroid ATP content (Fig. S6b). Additionally, we took microscopy images of the spheroids before and after a 24 h metabolism measurement in our hanging-drop setup (Fig. S6c). These measurements show that nutrient starvation leads to lower ATP accumulation in the spheroid and to stunting of spheroid growth. Finally, experimental values of q_S_ were obtained by fitting the measurement model via equation (19).

By fitting the value of $\tilde S$ for a given estimated value of D_S_ (Fig. 5a), we found the value of $\tilde S$ for the spheroids used in each experiment. From the spheroid model, we know that the glucose distribution within the spheroid is entirely described by $\tilde S$ in the solution to Eq. (8) (c_g_(r) in Supplementary Information S1). We plotted the initial radial distribution of glucose through the spheroid immediately after the target 150 μM glucose flow was stopped, as shown in Fig. 5b. This c_g_(r) is shown for the cases of a small spheroid (285 μm diameter) with a spheroid number of 1.7 and a large spheroid (453 μm diameter) with a spheroid number of 2.7 (Fig. 5b). The heterogeneous glucose distribution translates to a lower spheroid metabolism than if the entire spheroid volume was consuming glucose at a constant rate (Fig. 5c). For the highlighted small and large spheroids, their relative metabolic activity ratios are 85% and 70%, respectively.

## Discussion

We will first discuss experimental considerations, mainly (i) the selection of the cell line and medium for our biological model; (ii) the improvements to our integrated hanging-drop biosensor design; and (iii) how we handled biosensor sensitivity drift. Then, we will discuss the current responses, i.e., measuring H_2_O_2_ concentrations in the drops to extract information about glucose dynamics, mainly (iv) the complementary information provided by our bare-hydrogel-coated electrodes and (v) our interpretation of the values of our exponential parameter fitting. We will discuss (vi) glucose consumption by the spheroid and biosensor. Finally, we will conclude with (vii) how our measurement model can be applied in broader scenarios and how the spheroid number fits in the broader scheme of the growing usage of 3D biological models in the field of substance testing.

### Biological model selection

To validate our measurement model, we had to satisfy the conditions to probe first-order kinetics per our biosensor and spheroid models—ensuring low-glucose concentrations throughout the spheroid. Therefore, we ran metabolism measurements at low-glucose concentrations (150 μM). Applying a flow with this $c_g^ \ast$ established an initial glucose distribution (c_g_(r) through the spheroid (Fig. 5b). Experimentally, this required a cell line that remained metabolically active in a simplified medium formulation and at low-glucose concentrations.

#### Cell line

The selected cell line HCT116 allowed the formation of well-defined spheroids with uniform diameters (Fig. S6a). The resilience of the cell line allowed culturing in simplified medium formulations with a proportionally minimal impact on viability and ATP content (Fig. S6b). Since the majority of ATP production in cancer cells comes from aerobic glycolysis,^24^ i.e., the Warburg effect, we expected an ATP content decrease that was proportional to the glucose content decrease. The ATP measurements in Fig. S6b show that a ~75-fold reduction in glucose levels from more than 11 mM in our maintenance medium to 150 μM in our measurement medium induced only a 30-fold reduction in ATP levels in spheroids cultured in our chip. Finally, H_2_O_2_ concentrations of up to 200 μM did not affect HCT116 viability^25^. These findings confirmed that starvation—not oxidative stress due to H_2_O_2_ build-up—caused the effect on HCT116 ATP content. These starvation conditions mean that our results were representative of cells in first-order consumption kinetics, which was the premise of our spheroid model.

#### Medium selection

Selection of a phosphate buffer instead of a complex medium formulation obviated the nonspecific detection of other molecules by the electrochemical biosensors while ensuring that glucose was the singular energy source for cells. Additionally, the choice of a buffer with a low-glucose concentration (150 μM) was made (1) to ensure operation of the biosensor in its linear range, and (2) to guarantee first-order kinetics in both the spheroid glucose consumption and the glucose conversion in the functionalized hydrogels. Starvation levels of glucose also ensured that the spheroids did not grow excessively, therefore maintaining a constant size during the course of an experiment (Fig. S6c).

### Hanging-drop-integrated glucose biosensor design

Compared to our previous hanging-drop biosensors^17^, the new biosensor and microfluidic system is fully integrated. The new design features several key improvements that we elaborate on in the following paragraphs.

To increase drop stability, we reduced the hanging drop aperture diameter to 3 mm. This radius reduction increased the surface tension—i.e., increased the capillary pressure—which facilitated chip manipulation and allowed higher flow rates. Additionally, the resulting lower hanging-drop volume enabled us to measure glucose consumption faster.

To precisely control the glucose concentration with the flow, we reduced the number of hanging drops in series. This is particularly important when measuring consumption by larger spheroids. These larger spheroids cause a slight decrease in glucose concentration for each successive hanging drop in series, which would build up to a significant decrease for four drops in series—especially with our low-glucose application (Fig. S1). The effect of having multiple drops in series can also be appreciated by looking at the current signal of bare-hydrogel-coated electrodes, which is directly proportional to the bulk H_2_O_2_ concentration, in drops downstream from GOx-functionalized electrodes (Fig. S3). In this case, the initial current at downstream bare-hydrogel-coated electrodes is higher than that at upstream bare-hydrogel-coated electrodes. This current increase is due to H_2_O_2_ advective transport from upstream GOx-functionalized electrodes to downstream bare-hydrogel-coated electrodes. This finding further argues for limiting the number of drops in series.

To increase throughput, we implemented two parallel lines (Fig. 2a). Therefore, we have the option to run the chip either with a single condition throughout or with two separate conditions. Here, all experiments are shown with a single inlet for the four drops—meaning that odd drop numbers denote “upstream” drops, and even drop numbers denote “downstream” drops (Fig. 4a).

To minimize the diffusion distance between the spheroid and biosensor, we decreased the chip height. The channels were 250 μm in height, whereas the main hanging-drop compartments were 500 μm in height (Fig. 2c). The resulting hanging-drop compartment volume reduction also allowed us to measure spheroid glucose consumption faster. The changes reduced the medium-PDMS interface area by more than half, reducing potential molecule absorption and rerelease. Optical access to both the spheroid and the electrodes was always possible.

To increase the stability of the electrode functionalization layer, we reduced the thickness of the SU-8 rings guiding hydrogel deposition to 5 μm (Fig. 1b). Using a high-precision manual drop dispenser, deposition repeatability was also improved (Fig. S2d).

We oriented biosensor pairs perpendicular to the flow (Fig. 2a, c). This change limits the unwanted transport of H_2_O_2_ across electrodes within a hanging-drop compartment during flow. This also effectively synchronized the current signal through all upstream and downstream electrodes in two “batches” separated by 42 seconds (Fig. 3a inset and Fig. S1).

### Biosensor sensitivity

As evidenced by a reduction in sensitivity over multiple experiments, the ability of the functional sensor coating to convert glucose degrades over time (Fig. S8). This expected degradation was due to the deactivation of GOx by H_2_O_2_ over long-term experiments^26^. This degradation also reduces the generated H_2_O_2_, effectively increasing the glucose LoD because the H_2_O_2_ LoD (Fig. S4b) limits sensing. The typical LoD of a freshly coated biosensor can be as low as 0.4 ± 0.1 μM and will increase up to tenfold as the chip is continuously reused (Fig. S8).

Our fluidic setup and measurement strategy allowed us to cope with this degradation by performing sensitivity measurements before and after every measurement. The application of higher glucose concentrations to explore the linear range of our biosensor amplified this glucose oxidase deactivation, as evidenced by the rapidly decreasing current for concentrations over 300 μM (Fig. S2c, d). Therefore, we avoided probing the linear range of our biosensors during metabolism measurement experiments and performed only 3-point flow calibrations.

### Bare-hydrogel-coated electrode measurements

The advantage of bare-hydrogel-coated electrodes is their ability to directly measure H_2_O_2_ concentrations and transport dynamics in the hanging drops. A bare-hydrogel-coated electrode in a hanging drop with no spheroid (Fig. 4b) showed how the current dynamics on the bare-hydrogel-coated electrode were similar to the dynamics at the GOx-functionalized electrode. Additionally, bare-hydrogel-coated electrodes in hanging drops with spheroids (Fig. 4c) showed that H_2_O_2_ concentrations were much lower in the presence of a spheroid, as there was competitive glucose consumption by the spheroid. In the case of a spheroid-containing drop, glucose was rather quickly consumed (~20–40 min), and the GOx enzyme could no longer generate significant amounts of H_2_O_2_. In this scenario, the platinum electrodes were eventually able to appreciably oxidize H_2_O_2._ The current eventually decreased after more than 15 h (Fig. S4a), showing the electrode-oxidation effect described in Fig. 1c.

### Interpretation of the biosensor measurement model

In certain cases, the fitted parameters (Table 1) can take complex conjugate values, with the proportional coefficients $\bar a = c$ and exponential coefficients $\bar b = d$. In these cases, we demonstrated that the analytical solution remained real (Supplementary Information S4). Comparing the fitting parameter solutions in Table 1 to the results in Fig. 4d, we noticed that only current curves that showed an initial upward trend (e.g., an H_2_O_2_ build-up) featured complex parameter solutions.

We interpret these complex coefficients by drawing an analogy between circuit analysis and the analysis outlined in this paper. Complex numbers in the coefficients imply a delaying component in the differential equation system defined for glucose (13) and H_2_O_2_ (14) that was not clearly reported in our model. This delaying component can be interpreted either as a build-up of H_2_O_2_—i.e., charge accumulation—in the hanging-drop system or as a diffusion-caused time-delay component—i.e., a phase change. Our interpretation has two implications: (1) the real part of the exponential coefficients still provides information on consumption dynamics (q_S_), i.e., an exponential decay of current, as glucose and H_2_O_2_ are fully consumed; (2) the imaginary part of the exponential coefficients provides information on a diffusion or accumulation component, which induces a delay in the decaying current.

The most important aspect of our model is that it inherently considers H_2_O_2_ enzymatic conversion by our GOx-functionalized electrodes in addition to diffusion into—and subsequent accumulation within—the hanging-drop compartment. These diffusion and accumulation characteristics are evidenced by the fact that we still see a sensor signal, even when all glucose has been consumed (Fig. 4d in drops 5, 6, and 7). The corresponding measured current shows a delay in the signal dynamics. Moreover, we see that the glucose fitting curves (Fig. 4d blue dotted lines) behave as expected in all cases. No-spheroid case: We observed a slow decrease in glucose concentration, followed by a starker decrease as the glucose diffused into neighboring drops. We also observed that the glucose concentration decrease was more pronounced for empty drops neighboring drops with large spheroids (drop 8) than for empty drops neighboring drops with small spheroids (drop 1). This observation showed that minimizing drop serialization is important for low-concentration applications. We suggest that two serial drops constitute a good balance between experimental replicates and glucose diffusion from empty drops. Small-spheroid case: We observed a steady decrease in glucose over the course of the experiment. This decrease was uniform across the three drops 2, 3, and 4, as the spheroids were of relatively similar size (Fig. S6a). Large-spheroid case: We observed a stark initial decrease in glucose, as the spheroid rapidly consumed most glucose available to it.

### The biosensor and spheroid numbers

Equations (5) and (9) feature two dimensionless numbers. Both of these numbers are characteristic of the solution to the diffusion-reaction Eq. (2) and (4a). They are variants of the second Damköhler number, which is the ratio of the chemical reaction rate to the diffusive mass transfer rate in a system.

#### Biosensor number

This number describes the distribution of glucose throughout the hydrogel.

21 $$\tilde B = H\sqrt {\frac{{v_g}}{{D_g}}}$$

When the dimensionless number is much lower than 1 $\left( {\tilde B \ll 1} \right)$, the entire thickness of the hydrogel is metabolically active. In practice, this holds when $\tilde B\, < \,0.01$ and results in a uniform glucose concentration throughout the hydrogel. When it is much higher than 1 $\left( {\tilde B \gg 1} \right)$, only the surface of the hydrogel is metabolically active. In practice, this holds when $\tilde B\, > \,100$. and results in a null glucose concentration through most of the hydrogel bulk.

The value of $\tilde B$ affects the dynamics of H_2_O_2_ production and the measured current from equation (6). During a typical experiment, D_g_ through the hydrogel and H remain constant for a given biosensor functionalization. However, the first-order glucose kinetic constant of GOx (v_g_) in the hydrogel varies over time due to enzyme deactivation by H_2_O_2_^26^. Therefore, a change in q_E_ per Eq. (5) during an experiment can be attributed to a change in enzymatic activity v_g_. Depositing a thicker hydrogel would increase $\tilde B$. and, therefore, increase biosensor sensitivity b by increasing the overall enzymat conversion of glucose q_E_, per Eq. (7). However, as we increase $\tilde B$, there are diminishing returns for the sensitivity increase due to glucose depletion at the hydrogel surface. In our case, ${{{\tilde{\mathrm B}}}}$ was ~0.25 (Fig. S8), which provided good sensitivity while maximizing the overall hydrogel activity.

#### Spheroid number

This number describes the distribution of glucose consumption through the spheroid.

22 $$\tilde S = R\sqrt {\frac{{v_S}}{{D_S}}}$$

When the dimensionless number is much lower than 1 $\left( {\tilde S \ll 1} \right)$, the entire spheroid is homogeneously metabolically active. When it is much higher than 1 $\left( {\tilde S \gg 1} \right)$, the spheroid will feature only a metabolically active shell (Fig. 5b). The effective glucose diffusion coefficient through the spheroid (D_S_) remains constant throughout experiments. During an experiment containing multiple metabolism measurements, we showed that R remained constant (Fig. S6c). Therefore, any change in q_S_ defined per Eq. (9) over the course of a single experiment—i.e., over multiple measurements—can be attributed to either (i) a change in the first-order glucose kinetic constant of cellular consumption (v_S_) or (ii) a spatial change in v_S_, as cell populations at the core of the spheroid experience necrosis and stop consuming glucose. Over multiple experiments, spheroids experienced identical initial culture conditions. Therefore, the first measurement in an experiment should feature identical initial v_S_ values.

## Conclusion

With this work, we performed thorough characterization and modeling of our hanging-drop-integrated biosensors. For this purpose, we distilled our experimental approach to strictly necessary elements that can be described with a model that can be solved analytically. We chose a straightforward single-layer functionalization method that generated current curves that could be fitted with a sum of two exponentials per equation (19). This functionalization was combined with blank-hydrogel electrodes that provided a direct readout of H_2_O_2_ levels in the hanging drops. We observed that fitting the current results with a sum of two exponentials seemed to fully capture the current dynamics during metabolism measurements. The simplicity of this fitting strategy combined with its promising results highlights that this is a powerful method to study concentration dynamics in a closed system. Ultimately, such a method could be implemented to estimate parameters in a live measurement, as it requires no initial parameter guesses or user input for fitting.

We complemented the characterization of our hanging-drop-integrated biosensors with our spheroid model. The purpose of the spheroid model was to provide insights into the concentration distribution within a spheroid from an external measurement. The derivation of the spheroid number shown in this work limits its validity to first-order reaction kinetics, i.e., to low-concentration applications. In the case of zeroth-order or Michaelis–Menten kinetics, an analysis of transport and consumption kinetics within the spheroid, analogous to our analysis, must be established.

However, the first-order kinetics assumption typically applies to most substance testing applications, where low concentrations are commonly used. Knowing the spheroid number of a specific compound interacting with a 3D tissue model is crucial for biological applications. As 3D tissue models become ubiquitous in substance testing, the assessment of the proportion of metabolically active cells within a spheroid will be necessary to correctly interpret experimental results. Our model and measurements show that the proportion of metabolically active cells can vary from a fully metabolizing spheroid to only cells located in an outer shell (Fig. 5c). Therefore, the spheroid number could provide a rapid and straightforward parameter for interpreting the in vitro results of 3D tissue models. Our developed method will facilitate translating results obtained with 3D tissue models from in vitro to in vivo.

## Materials and methods

To achieve reliable chip operation and reproducible results, the conditions in the liquid phase around the 3D biological model, in the microfluidic channels, and at the biosensors needed to be tightly controlled. Each metabolism measurement experiment was executed using a dedicated setup including a microscope, flow pumps, and a potentiostat (Fig. S2a).

### 3D biological model

#### Spheroid formation

The HCT116 cancer cell line (Sirion Biotech, Martinsried, Germany) was cultured using standard protocols, with a standard cell culture medium comprising RPMI-1640 (Gibco, Fisher Scientific, Illkirch Cedex, France), supplemented with 10% fetal bovine serum (FBS, Sigma–Aldrich, Buchs, Switzerland), and 1% penicillin and streptomycin (Sigma–Aldrich, Buchs, Switzerland), herein named “maintenance medium”. Cells were seeded 3 days prior to a metabolism measurement experiment to obtain uniform and well-defined spheroids of diameters varying between 250 and 550 μm (Fig. S6a). We controlled the diameters of the spheroids by seeding 150 or 900 HCT116 cells in the wells of a Nunc® 96-well, Nunclon Delta-treated, U-shaped-bottom microplate (Thermo Fisher Scientific, Reinach, Switzerland). The cells were subsequently spun down at 250 × *g* for 5 min to ensure that they formed a singular spheroid per well.

#### Spheroid characterization

Before and after a metabolism measurement experiment, we imaged the spheroids to assess their morphology. We also performed ATP measurements to control spheroid metabolism over time in and out of the hanging drop setup. ATP measurements were performed with the CellTiter-Glo® 3D Cell Viability Assay (Promega AG, Dübendorf, Switzerland). Imaging in the U-bottom 96-well microplate was performed with a plate scanner (Cell3iMager, SCREEN SPE Germany GmbH, Ismaning, Germany). Imaging in the hanging-drop setup was performed with an inverted wide-field microscope (Leica DMI6000B, Leica Microsystems, Switzerland) with a 10× lens.

#### Cell culture medium selection

The HCT116 cancer cell line was cultured using maintenance medium. However, for the metabolism measurement experiments, we used a cell culture medium with a minimal formulation. This medium must preserve spheroid integrity, minimize sensor noise, and allow the precise control of nutrient sources. We supplemented DPBS (with Mg^+^ and Ca^+^) buffer (14040091, Gibco, Thermo Fisher Scientific, Reinach, Switzerland) with glucose (G8270, Sigma–Aldrich, Buchs, Switzerland). A glucose concentration of 150 μM was used for target-range calibration and 1.5 mM for linear-range assessment. The buffer, target, and linear-range media are referred to as “measurement media”. The viability of spheroids in the measurement medium with 150 μM of glucose was compared to a control in the maintenance medium by measuring the ATP contents of spheroids of various sizes before and after a metabolism measurement experiment (Fig. S6b).

### Integrated hanging-drop biosensor

The hanging-drop biosensor system^17^ consisted of three parts: (1) a platinum-patterned glass substrate featuring a reference, counter, and several working electrodes, (2) a microfluidic channel network, and (3) the hydrogel for electrode functionalization.

#### Electrode fabrication

A microfabrication methodology nearly identical to the one previously published by Misun et al. was used^17,27^. This protocol entailed creating a masking layer with a lift-off resist and a positive-tone resist on a silicon wafer, which was used to pattern a subsequently deposited platinum trace with a lift-off process. Afterward, traces were passivated with a silicon nitride layer. After using a positive-tone resist to shield the traces, reactive ion etching was used to selectively expose the electrodes and connector pins. The result was a glass electrode chip that could be directly connected to a card reader fitting within a microscopy stage-top incubator (Fig. 2e).

Three main changes were implemented for this publication. (1) The electrode layout was changed to include a counter electrode in each hanging-drop compartment. This change gives more freedom when designing the microfluidic channel routing from one compartment to another. (2) The photolithography masks were printed on transparent foil masks (Micro Lithography Services Ltd, Essex, UK). This change allowed faster and cheaper electrode prototyping. (3) The thickness of the SU-8 rings—used as a phase guide for the hydrogel—was reduced from 50 to 5 µm. This change allowed a thinner and more stable hydrogel layer to be deposited, thus reducing the limit of detection and increasing the sensitivity of the biosensor.

Prior to assembly, the reference electrode on the glass electrode chips must be coated with Ag/AgCl. We used an Ag/C/AgCl paste (106-12, Creative Materials, Inc., Ayer, USA) diluted in a solvent (113-12, Creative Materials, Inc., Ayer, USA) at a 4:1 weight ratio. The solution was dispensed on the reference electrode using a droplet dispenser (Ultimus Präzisionsdosiergeräte, Nordson, Vilters, Switzerland) at a dispensing pressure of 5 bar and a flow-back pressure of 6 kPa for 2 ms. The paste was subsequently cured on a hot plate at 180 °C for 1 h.

#### Microfluidic channel fabrication

We developed a straightforward soft lithography protocol allowing for fast prototyping of a thin PDMS substrate with through-holes for the electrodes and thicker inlets for the tubing connectors (Fig. S7). To form the PDMS (Sylgard 184, Dow Corning GmbH, Wiesbaden, Germany), we used a 3D-printed mold (Protolabs, Feldkirchen, Germany—Accura SL5330 with a xy-resolution of 200 μm and z-resolution of 50 μm) as the bottom mold and laser-cut acrylic (Plexiglas GS, Evonik Performance Materials GmbH, Darmstadt, Germany) slides as the top mold. We designed the laser-cut acrylic slides to reproduce the exact dimensions of the glass electrode chip and to be firmly locked in place with the help of pillars on the 3D-printed mold. These two design features of the slides allowed hassle-free alignment of the glass electrode chip to the microfluidic channels during glass-PDMS bonding.

To ensure proper resolution of the features, the PDMS degassing and curing processes were each split into two steps. (1) After casting PDMS on the 3D-printed mold, the first degassing step (10 min) was completed to ensure proper structure resolution. (2) By slowly lowering the laser-cut acrylic slides, most air bubbles trapped between the uncured PDMS and the slide were pushed out. A weight was placed on the slides, and a second degassing step (45–60 min) was performed to remove any remaining trapped air bubbles. (3) The first curing step (1 h at 80 °C or 4 h at 65 °C) ensured that most of the PDMS was cured. (4) Since PDMS curing is an oxygen-dependent process and since acrylic is not oxygen permeable, removing the laser-cut acrylic slide exposed uncured PDMS. The second curing step (1 h at 80 °C or 4 h at 65 °C) ensured that all of the PDMS was properly cured.

Before demolding, we verified that the hanging-drop compartment through-holes were open by scraping PDMS residues with a scalpel. Afterward, the microfluidic channels were ready to be demolded and bonded onto the glass electrode chip with an oxygen plasma (40 W, 30 s, 6 mbar O_2_). The final step was to punch inlets and outlets in the thick parts of the PDMS with a 0.5 mm-diameter hole puncher.

#### Electrode functionalization

Working electrodes were functionalized using the same glutaraldehyde-based method as in our previous publication^17^, allowing analyte concentration measurements in each hanging-drop compartment. Each compartment hosted one bare-hydrogel-coated electrode and one GOx-functionalized electrode. Two drop compartments featured an extra bare-hydrogel-coated platinum electrode that served to measure the extent of diffusion between electrodes. The functionalization was optimized to improve its stability and uniformity. The hydrogel was formed with a base of deionized water, bovine serum albumin (Sigma–Aldrich, Buchs, Switzerland), and glutaraldehyde 25% (Sigma–Aldrich, Buchs, Switzerland). The functional component of the hydrogel was GOx (Sekisui Diagnostics, Kingshill, UK). The final recipes and protein ratios are summarized in Table 2. The droplet dispenser was used to functionalize electrodes at a dispensing pressure of 0.6 bar and a null flow-back pressure for 0.5 ms.

**Table 2:** List of materials and recipes to form the hydrogel bulk for electrode functionalization.

		Bovine serum albumin	Glucose oxidase	Deionized water	Glutaraldehyde
	Weight ratio (%)	96%	88%	100%	25%
**ID**	**Hydrogel recipes**	**mg**	**mg**	**µL**	**µL**
GOx	Glucose Oxidase Hydrogel	5.5	6.8	220	40
BLK	Blank Hydrogel	10	0	235	20

### Experimental setup

Performing a metabolism measurement required several electronic and microfluidic interacting components. The following three elements were needed: (1) a potentiostat to monitor the currents generated at the working electrodes, (2) precision-grade syringe pumps and flow manifolds to actuate the medium flow and composition through the device, and (3) an inverted microscope to control the hanging-drop size and shape. The experimental setup is shown in Fig. S2a. The general fluidics and drop-volume control strategies were outlined in detail in a previous publication^28^.

#### Potentiostat readout

The working principle (Fig. 2c) of our biosensor was based on a three-electrode setup with 18 working electrodes. A custom potentiostat^19^ was previously developed in our laboratory that allows us to readout all working electrodes simultaneously. The potentiostat operates at a sampling frequency of 90,726 Hz. A cascaded integrator-comb filter of third order with a decimation ratio of 64 was combined with an exponential moving average with a 255 ratio to reduce the sampling frequency to 5.56 Hz. The working electrode voltage was set to 0.65 V versus our Ag/AgCl reference electrode. To connect the biosensor to the potentiostat, we used a card guide soldered onto a custom printed circuit board that fits within a Nunc™ OmniTray™ single-well plate (Thermo Fisher Scientific, Reinach, Switzerland). We interfaced the potentiostat on the control computer via a USB cable and LabView. Before analysis, the current readout passed through a low-pass filter. As we were interested in current changes occurring at a time scale of minutes, knowing our experimental sample rate of 5.56 Hz, we set the filter window size (order) to 1025 with a cutoff frequency of 16.7 Hz. The raw data were filtered using a Blackman window vector and the FIR1 function available in MATLAB, which effectively removed high-frequency artifacts.

#### Fluidics control

Metabolism measurement experiments required a relatively minimal “measurement medium”, but HCT116 spheroids thrive in their “maintenance medium”. The use of syringe pumps (neMESYS, Cetoni GmbH, Korbussen, Germany) and custom flow manifolds enabled switching from the “measurement medium” to the “maintenance medium” within the setup to repeat metabolism measurements over multiple days. The flow manifolds allowed us to control the concentration of glucose by adjusting the relative flow rates of the measurement media (Fig. S2b). From our numerical simulations (Fig. S1 and Movies S1 and S2), we established that exchanging the medium in the entire drop at a flow rate of 5 μL min^−1^ per line—for a total of 20 μL min^−1^ through the chip—took 7.5 min for 800 μm-high drops. To ensure a steady current plateau for each target calibration concentration, we perfused each target concentration for 15 min. Whenever a syringe pump was activated after a longer period of deactivation, this time was increased to 20 min to ensure proper mixing (Fig. 3a and Fig. S2b, c timing for 0, 75, and 300 μM). Ultimately, we performed a 3-point flow calibration with 0, 75, and 150 μM glucose-spiked buffer (Fig. 3a).

#### Drop size control

The fluidic network consisted of four parallel channels with two hanging drops each, supplied via one or two inlets. We used continuous imaging of a free-floating 10 μm-thick SU-8 ring that sedimented to the bottom of the hanging drop to control the height of a single hanging drop^29^. This continuous imaging was performed with an inverted microscope using a 10x objective. A feedback loop adjusted the flow rate to keep the SU-8 ring at a constant focus height. This feedback loop was implemented in the open-source YouScope software as a proportional integral controller (PIC) under the “Droplet Based Microfluidics” job^30^. We ignored the integral part of the PIC by setting a large value of 10 days for the integral constant. The proportional part of the PIC was set to 10 min. The height-to-volume ratio of a hanging drop was set using the following equation.

23 $$\frac{h}{V} = \frac{8}{{\pi N \left( {a^2 + 4h^2} \right)}}$$

This yielded 30 μm/μL for our 600 μm-high (h) and 3 mm-diameter (a) drops within a microfluidic network consisting of 8 drops (N).

### Experimental workflow

Each experiment comprised (1) a settling curve, (2) sensor calibrations, (3) metabolism measurements, and (4) spheroid maintenance.

(1) Settling curve: A 1 h settling curve was recorded once at the beginning of every experiment.

(2) Sensor calibrations: Calibrations involved perfusing two to three types of measurement medium: a plain buffer (DPBS with Mg^+^ and Ca^+^), a buffer spiked with the target starvation glucose levels (+150 μM glucose), and an optional third buffer spiked with higher glucose levels (+15 mM glucose). A typical sensor calibration started with a sensitivity measurement (Fig. 3c). This sensitivity measurement included a minimum of three 20 min concentration plateaus (0, 75, and 150 μM) ramping from the plain buffer up to the target starvation glucose concentration. We optionally added ramp steps up to a higher glucose level (0.3, 0.75, 1.2, and 1.5 mM) to determine the linear range (Fig. 3c). Sensor calibrations were performed once immediately before and once immediately after each metabolism measurement.

(3) Metabolism measurements: Measurements started with a step decrease to the plain buffer, followed by a step increase to the target starvation glucose level. Afterward, all flow was stopped, and the spheroids were allowed to consume the glucose present in the hanging drop. Metabolism measurements were performed several times during an experiment.

(4) Spheroid Maintenance: Maintenance involved perfusing maintenance medium. Spheroid maintenance ran in between metabolism measurements.

An example of an experimental flow scheme is shown in Fig. S2. The ratio between various syringe pumps is modulated to ensure a continuous flow while controlling the prescribed glucose concentration through the hanging-drop compartments during the settling curve, sensor calibrations, and spheroid maintenance. The associated measured drop volume is also shown and was calculated from the free-floating SU-8 height position measurement. This demonstrates the control that we have over the concentration in the hanging drop and the drop stability.

## Supplementary information

Supplemental Material

Supplementary Movie 1

Supplementary Movie 2

## Data Availability

The experimental data supporting the findings in this work and the custom code used to analyze the data are available from the corresponding author on request.
