# Supplementary material for: Modeling and measuring glucose diffusion and consumption by colorectal cancer spheroids in hanging drops using integrated biosensors
Source: Microsyst Nanoeng. 2022 Feb 1;8:14. doi: 10.1038/s41378-021-00348-w (PMC8803859; doi:10.1038/s41378-021-00348-w)
Supplement: Supplementary file 1 — Supplemental Material [file 41378_2021_348_MOESM1_ESM.docx]

# Modeling and Measuring of Glucose Diffusion and Consumption by Colorectal Cancer Spheroids in Hanging Drops using Integrated Biosensors: Supplementary Information

Nassim Rousset* (nassim.rousset@bsse.ethz.ch)
Rubén López Sandoval (rsandoval@student.ethz.ch)

Mario Matteo Modena (mario.modena@bsse.ethz.ch)
Andreas Hierlemann (andreas.hierlemann@bsse.ethz.ch)
Patrick M. Misun (patrick.misun@bsse.ethz.ch)

Affiliation: ETH Zürich, Department of Biosystems Science and Engineering, Bio Engineering Laboratory, Mattenstrasse 26, Basel CH-4058, Switzerland

*Corresponding author:

Nassim Rousset
+41 61 387 31 83
nassim.rousset@bsse.ethz.ch

1. Biosensor and spheroid model solutions

*Biosensor model:* 1D steady-state solutions for glucose and hydrogen peroxide in a hydrogel under a flow condition (constrained concentrations at hydrogel-medium boundary) are given as follows:

|  | $c_{g}\left( x \right)=c_{g}^{*}\frac{\cosh\left( \tilde{H}\frac{x}{H} \right)}{\cosh\left( \tilde{H} \right)}$ | (24) |
| --- | --- | --- |
|  | $c_{h}\left( x \right)=c_{g}^{*}\frac{D_{g}}{D_{h}}\frac{1-\cosh\left( \tilde{H}\frac{x}{H} \right)-\frac{x}{H}\left( 1-\cosh\left( \tilde{H} \right) \right)}{\cosh\left( \tilde{H} \right)}$ | (25) |

The flux of glucose coming into the hydrogel is given by Fick’s first law (and compared to the average consumption by the hydrogel):

|  | $D_{g}\left. \frac{\partial c_{g}\left( x \right)}{\partial x} \right\vert_{x=H}=c_{g}^{*}\sqrt{D_{g}v_{g}}\tanh\left( \tilde{H} \right)=q_{E}Hc_{g}^{*}$ | (26) |
| --- | --- | --- |

Therefore, when the flow of glucose is stopped, this can be considered as a new boundary condition at the hydrogel boundary. This is regarded as a Robin boundary condition, as the derivative of the function at the boundary is proportional to the function itself. This can be used as an alternate boundary condition to include diffusion within the measurement model.

*Spheroid model:* The 1D steady-state solution for glucose in a spheroid under a flow condition (constrained concentration of glucose at the spheroid-medium boundary) is given as follows:

|  | $c_{g}\left( r \right)=c_{g}^{*}\frac{R}{r}\frac{\sinh\left( \tilde{S}\frac{r}{R} \right)}{\sinh\left( \tilde{S} \right)}$ | (27) |
| --- | --- | --- |

The flux of glucose coming into the spheroid is given by Fick’s first law (and compared to the average consumption by the spheroid).

|  | $D_{g}\left. \frac{\partial c_{g}\left( r \right)}{\partial r} \right\vert_{r=R}=\frac{D_{g}c_{g}^{*}}{R}\left( \tilde{S}\coth\left( \tilde{S} \right)-1 \right)=\frac{q_{S}R}{3}c_{g}^{*}$ | (28) |
| --- | --- | --- |

As a reminder, the average consumption by the spheroid is given by:

|  | $q_{S}=3\frac{D_{S}}{R^{2}}\left( \tilde{S}\coth\tilde{S}-1 \right), \tilde{S}=\sqrt{\frac{v_{S}}{D_{S}}}$ | (29) |
| --- | --- | --- |

Using Robin boundary conditions – same as with the biosensor model – can be used as an alternate boundary condition to include diffusion within the measurement model.

1. Diffusion through the biosensor

We characterized hydrogen peroxide (H_2_O_2_) diffusion in our chip with an experiment without spheroids. A special GOx-functionalization of the biosensor (Figure S3) allowed us to quantify the rate of diffusion across the hanging drops. The null-current value of all electrodes was set by applying a flow with a buffer without glucose. Afterward, a flow with a buffer containing 300 μM of glucose was applied through the microfluidic chip. Finally, the flow was cut at time 0, and we measured the varying currents (Figure S3). This experiment was additionally modeled numerically using the COMSOL Multiphysics® v. 5.4 software (COMSOL AB, Stockholm, Sweden) with the Laminar Flow and Transport of Diluted Species physics implemented with the experimental enzymatic conversion rate of GOx-functionalized electrodes (Figure S1 and Movies S1 and S2).

*Cross-line diffusion:* This is defined as diffusion of species across two neighboring parallel drops (e.g. drop 1 is “cross-line” to drop 3). The 100-fold current decrease from drop 3 to drop 1 and from drop 4 to drop 2 is evidence that cross-line diffusion was negligible.

*Cross-drop diffusion:* This is defined as diffusion of species across two neighboring serial drops (e.g. drop 2 is “cross-drop” to drop 2). The 10-fold current decrease from drop 6 to drop 5 and from drop 7 to drop 8 is evidence that cross-drop diffusion was significant toward drops that do not contain GOx-functionalized electrodes. Comparing the bare-hydrogel-coated electrodes from drops 3 and 6 (Electrodes #3 and 14 respectively) shows that cross-drop diffusion did not significantly affect the signal within a drop that contained a GOx-functionalized electrode. This demonstrates how the normal experimental functionalization scheme, per Figure 2a, allowed us to neglect cross-drop diffusion.

*Cross-drop flow:* This is defined as flow-induced species transport across two neighboring serial drops. Bare-hydrogel-coated electrodes upstream from a GOx-functionalized electrode (Electrodes #5, 6), not within the same line as a GOx-functionalized electrode (Electrodes #1, 2, 17, 18) or within the same compartment (electrodes #3, 8 and 14) started with a current around the ~10 pA range. This shows that the flow initialization of the experiment was successful at setting a null H_2_O_2_ concentration in upstream drops. All bare-hydrogel-coated electrodes downstream from a GOx-functionalized electrode started with a current around the ~100 pA range. This shows that the flow initialization of the experiment brought a small amount of H_2_O_2_ from the upstream drops to the downstream drops.

*Cross-electrode diffusion:* This is defined as diffusion of species across electrodes in the same hanging-drop compartment (e.g. electrode 1 is “cross-electrode” to electrode 2). Bare-hydrogel-coated electrodes in drops 3, 4, 6, and 7 (Electrodes #3, 15, 14, and 8 respectively) showed that cross-electrode diffusion was significant. Including bare-hydrogel-coated electrodes in every drop allowed us to measure this diffusion and quantify H_2_O_2_ build-up in the hanging drop.

*Effect of multiple functionalized electrodes:* If two GOx-functionalized working electrodes are located in the same hanging-drop compartment, the enzymatic conversion of glucose, yielding H_2_O_2_, is increased in the compartment. This is evidenced by the increased signal coming from electrode #8, neighbored by two GOx-functionalized electrodes #7 and 9, when compared to electrode #3, neighbored by a single GOx-functionalized electrode #4.

1. Fitting strategy to find the parameters

Using linear algebra, we could fit the evaporation-corrected current without using “initial guesses”.

|  | $y\left( t \right)=i\left( t \right)\left( V_{0}-V_{t}t \right)=ae^{bt}+ce^{dt}$ | | (30) | |  |
| --- | --- | --- | --- | --- | --- |
|  | | $\begin{matrix} \int y\left( t \right)dt=\frac{a}{b}e^{bt}+\frac{c}{d}e^{dt}+k_{1}, & \iint z\left( t \right)dt=\frac{a}{b^{2}}e^{bt}+\frac{c}{d^{2}}e^{dt}+k_{1}t+k_{0} \end{matrix}$ | | (31) | |

With an integration constant $k$. We could add the two integrals and the temporal component with coefficients $A_{1-4}$. We factorized the $A_{1-4}$ components to isolate each exponential and temporal component.

|  | $A_{1}\int y\left( t \right)dt+A_{2}\iint y\left( t \right)dt+A_{3}t+A_{4}=y\left( t \right)$  $\left( A_{1}\frac{a}{b}+A_{2}\frac{a}{b^{2}} \right)e^{bt}+\left( A_{1}\frac{c}{d}+A_{2}\frac{c}{d^{2}} \right)e^{dt}+\left( A_{2}k_{1}+A_{3} \right)t+\left( A_{1}k_{1}+A_{2}k_{0}+A_{4} \right)=ae^{bt}+ce^{dt}$ | (32) |
| --- | --- | --- |

Since exponentials with various exponential coefficients are orthogonal, the previous sum in equation (32) allowed us to conveniently determine the exponential coefficients. By equating the terms multiplying the exponential $e^{bt}$ and $e^{dt}$ in equation (32), we found the value of the exponential coefficients $b$ and $d$ by finding the roots of the following polynomial.

|  | $\left. \begin{matrix} A_{1}\frac{a}{b}+A_{2}\frac{a}{b^{2}}=a \\ A_{1}\frac{c}{d}+A_{2}\frac{c}{d^{2}}=c \end{matrix} \right\}x^{2}-A_{1}x-A_{2}=0\left\{ \begin{aligned} x_{1}=b \\ x_{2}=d \end{aligned} \right.$ | (33) |
| --- | --- | --- |

In practice, by using the data, we could evaluate the following matrix using the pseudo-inverse of a matrix to find the coefficients $A_{1-4}$ and, in turn, find $b$ and $d$.

|  | $\left[ \begin{matrix} \begin{matrix} A_{1} \\ A_{2} \end{matrix} \\ \begin{matrix} A_{3} \\ A_{4} \end{matrix} \end{matrix} \right]=\left[ \begin{matrix} \begin{matrix} \sum y_{\text{data}}\Delta t & \sum\sum y_{\text{data}}\Delta t \\ \vdots& \vdots\end{matrix} & \begin{matrix} t & 1 \\ \vdots& \vdots\end{matrix} \end{matrix} \right]^{-1}\left[ \begin{matrix} y_{\text{data}} \\ \vdots\end{matrix} \right]$ | (34) |
| --- | --- | --- |

Replacing the found exponential coefficients into equation (30), we could then find the proportionality coefficients $a$ and $c$.

|  | $\left[ \begin{matrix} a \\ c \end{matrix} \right]=\left[ \begin{matrix} \exp\left( bt_{\text{data}} \right) & \exp\left( dt_{\text{data}} \right) \\ \vdots& \vdots\end{matrix} \right]^{-1}\left[ \begin{matrix} y_{\text{data}} \\ \vdots\end{matrix} \right]$ | (35) |
| --- | --- | --- |

This gave the least-squares fit with the experimental data for a sum of two exponentials. The least-squares fit of the evaporation-corrected current $y\left( t \right)$ was, therefore, calculated with the exponential coefficients $b$ and $d,$ found with equation (33), and with the proportionality coefficients $a$ and $c,$ found with equation (35).

1. Special cases for the found parameters

In the case that the solutions to $a$, $b$, $c$, and $d$ are real, the solution and its parameters simplify to the following:

|  | $y\left( t \right)=a\exp\left( bt \right)+c\exp\left( dt \right)\left\{ \begin{matrix} \begin{matrix} q_{e}=-b \\ q_{E}+q_{S}=-d \end{matrix} \\ \begin{matrix} m_{h}=\frac{c\left( \frac{b}{d}-1 \right)}{FAc_{g0}} \\ c_{h0}=c_{g0}\frac{\frac{a}{c}+1}{\frac{b}{d}-1} \end{matrix} \end{matrix} \right.$ | (36) |
| --- | --- | --- |

In the special case where $q_{e}\sim q_{E}+q_{S}$, equation (17) takes a special form. In this case, the fitting equation is slightly different.

|  | $y\left( t \right)=FAm_{h}\left( c_{h0}+c_{g0}q_{e}t \right)\exp\left( -q_{e}t \right)$ | (37) |
| --- | --- | --- |
|  | $y\left( t \right)=\left( a+ct \right)\exp\left( bt \right)$ | (38) |

In the case that the solutions to $a$, $b$, $c$, and $d$ are complex conjugates (i.e. $\bar{q_{e}}\sim q_{E}+q_{S}$), the solution and its parameters simplify to the following.

|  | $y\left( t \right)=\left\{ \begin{matrix} FAm_{h}\left( c_{h0}\cos\left( \mathfrak{I}\left( q_{e} \right)t \right)+c_{g0}\frac{\mathfrak{R}\left( q_{e} \right)}{\mathfrak{I}\left( q_{e} \right)}\sin\left( \mathfrak{I}\left( q_{e} \right)t \right) \right)e^{\mathfrak{-R}\left( q_{e} \right)t} \\ -iFAm_{h}\left( c_{h0}+c_{g0} \right)\sin\left( \mathfrak{I}\left( q_{e} \right)t \right)e^{\mathfrak{-R}\left( q_{e} \right)t} \end{matrix} \right.$ | (39) |
| --- | --- | --- |
|  | $\left. \begin{matrix} a=\bar{c} \\ b=\bar{d} \end{matrix} \right\}\to y\left( t \right)=2\left( \mathfrak{R}\left( a \right)\cos\left( \mathfrak{I}\left( b \right)t \right)\mathfrak{-I}\left( a \right)\sin\left( \mathfrak{I}\left( b \right)t \right) \right)\exp\left( \mathfrak{R}\left( b \right)t \right)$ | (40) |

Regardless of the form of the analytical solution, the fit $y\left( t \right)=a\exp\left( bt \right)+c\exp\left( dt \right)$ should converge.

1. Working voltage selection

We initially applied 0.65 V and 0.45 V at the working electrodes during separate experiments, measuring a sensitivity of 987 ± 46 nA mm^‑2^ mM^‑1^ and 607 ± 96 nA mm^‑2^ mM^‑1^ respectively, i.e. a 38% decrease. A voltage reduction by 0.2 V has been reported to entail a signal reduction of around 36%^21^. The higher target voltage of 0.65 V did not induce any observable effects on HCT116 spheroid viability and did not cause water electrolysis. Therefore, we applied 0.65 V for all metabolism measurements to obtain higher sensitivity with no measurable side effects.

1. Supplementary Figures


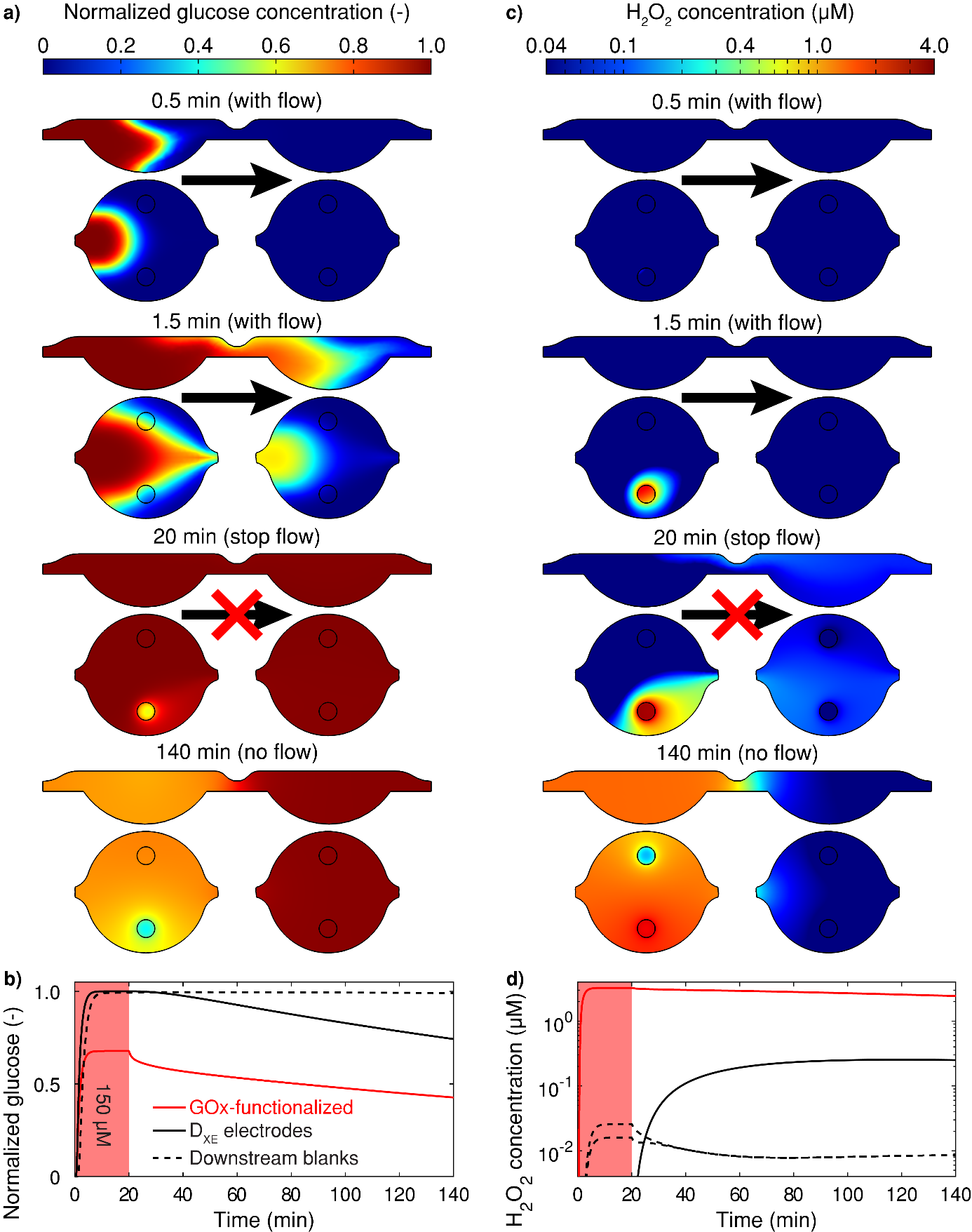


Supplementary Figure 1: **Numerical mathematical modeling of diffusion and consumption through the chip using COMSOL Multiphysics®.** All model results were obtained with an 800 μm hanging-drop height to be comparable to the experimental results of Figure S3. With smaller hanging-drop height, results show faster transport dynamics. All diffusion parameters were taken from literature (as cited within the manuscript), and the enzyme-consumption parameter was taken to represent the diffusion experiment results of Figure S3. a) Glucose-transport dynamics under flow seen from the colinear cross-section of the chip, and the top cross-section of the chip when normalizing to 150 μM glucose concentration. We did not correct for evaporation here. The glucose distribution in the upstream drop at 0.5 minutes was similar to that of the downstream drop at 1.5 minutes, which evidenced a 1-minute drop-to-drop delay. This timing delay was comparable to the 42 seconds measured in Figure 3a inset, which was done in a 600-μm-high hanging drop (i.e., we expect a faster liquid exchange dynamics). The flow was stopped at the 20-minute snapshot, allowing the GOx-functionalized electrode (bottom left) to perform enzymatic conversion of glucose, yielding H_2_O_2_. The 140-min snapshot (2 hours into stopping the flow) shows that the electrode in an empty drop does not appreciably reduce the glucose concentration in neighboring drops during a typical experiment. Movie S1 shows the dynamics of glucose changes through the chip over the snapshots shown in this figure. b) Summary of the normalized glucose dynamics when applying a 150 μM glucose concentration in our chip from a null concentration at 0 min. The average concentration at the GOx-functionalized (red line), cross-electrode (black line), and downstream cross-drop (dashed black line) electrodes is plotted. c) H_2_O_2_ transport dynamics under flow seen from the same points of view and time points as the glucose dynamics. A logarithmic scale is used to emphasize spatial H_2_O_2_ distribution. Downstream transport of H_2_O_2_ (e.g., drop 7 to drop 8) is particularly salient at the moment the flow is stopped (20 minutes). Over 2 hours into stopping the flow, H_2_O_2_ concentration still features a gradient throughout the drop, explaining the difference in current dynamics shown in Figure 4b. Movie S2 shows the dynamics of H_2_O_2_ concentration changes through the chip over the snapshots shown in this figure. d) Summary of the H_2_O_2_ dynamics similar to that shown for glucose. We found that 1% of the H_2_O_2_ was washed from the upstream GOx-functionalized electrode to the downstream electrode during flow. We show that the H_2_O_2_ concentration at a bare-hydrogel-coated electrode is expected to reach 10% of the concentration at a GOx-functionalized electrode. We show that the cross-drop H_2_O_2_ transfer is less than 1% of the H_2_O_2_ generated at the GOx-functionalized electrode.


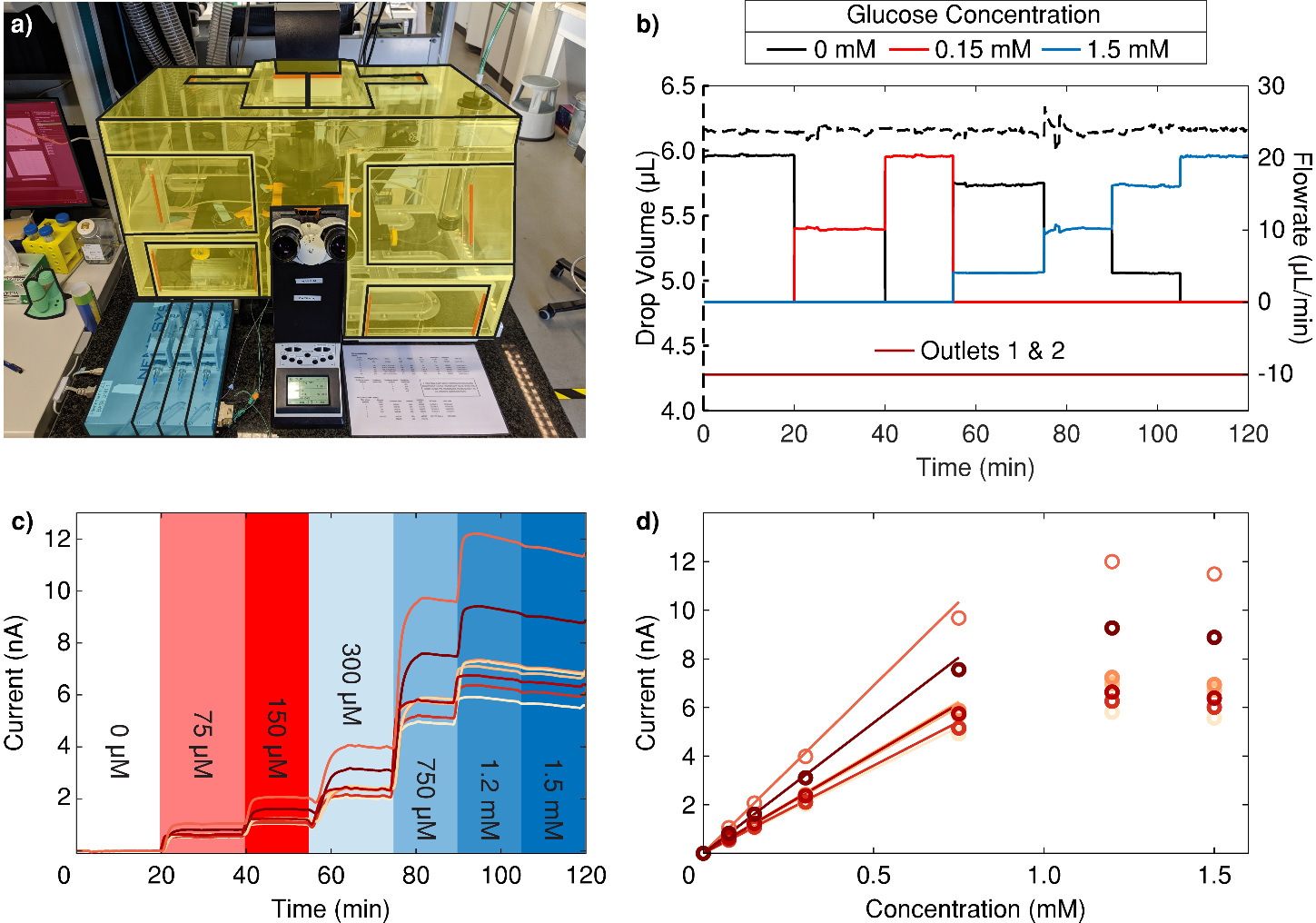


Supplementary Figure 2: **Process to generate a calibration curve.** The working electrodes feature a diameter of 0.4 mm, therefore, currents of 1 nA correspond to a current density of 2 nA mm^-2^. a) **Photo of the experimental setup required to operate the hanging-drop-integrated enzymatic biosensor.** Highlighted are the various components: the inverted wide-field microscope and its temperature-control incubation box, highlighted in yellow; the xyz-stage controller on the left, highlighted in green; the Nemesys precision syringe pumps, highlighted in blue; and the dedicated computer allowing interfacing with all the aforementioned components and the potentiostat (not in the image). b) **Example of the flow scheme required to control the concentration within our hanging-drop compartments.** Two outlet syringes are withdrawing liquid at a constant rate of 10 μL min^‑1^. Three types of solutions containing DPBS spiked with either 0 μM, 150 μM, or 1.5 mM of glucose were injected through the inlet at an average flow rate of 20 μL min^‑1^. The microscope monitors the drop height of one of the hanging drops and, with a feedback algorithm, keeps the hanging drop compartment volume constant (dashed black line). The relative ratio of the solution types allowed for various concentrations to be set in the hanging-drop compartments. The plotted flow scheme applies 0 μM for 20 minutes, 75 μM for 20 minutes, 150 μM for 15 minutes, 300 μM for 20 minutes, 750 μM for 15 minutes, 1.2 mM for 15 minutes, and 1.5 mM for 15 minutes. c) **Current response to the previous dosage scheme within eight hanging-drop compartments.** The GOx-functionalized enzymes of eight hanging-drop compartments are shown. The current response was reproducible from drop to drop and featured some deviations due to experimental variations in the deposition scheme from electrode to electrode. A 7.5 minutes transient could be observed as the new concentration flushed out the old. This was followed by a steady plateau for most target glucose concentrations. The H_2_O_2_ poisoning effect on the GOx enzymes can be seen by the rapid reduction in current response for high concentrations of glucose. d) **Calibration curve and linear range.** The data points (circles) show the current response to a target glucose concentration as measured in the previous panel c. The lines show the linear range of each electrode, which featured varying sensitivities due to experimental variations in the deposition scheme.


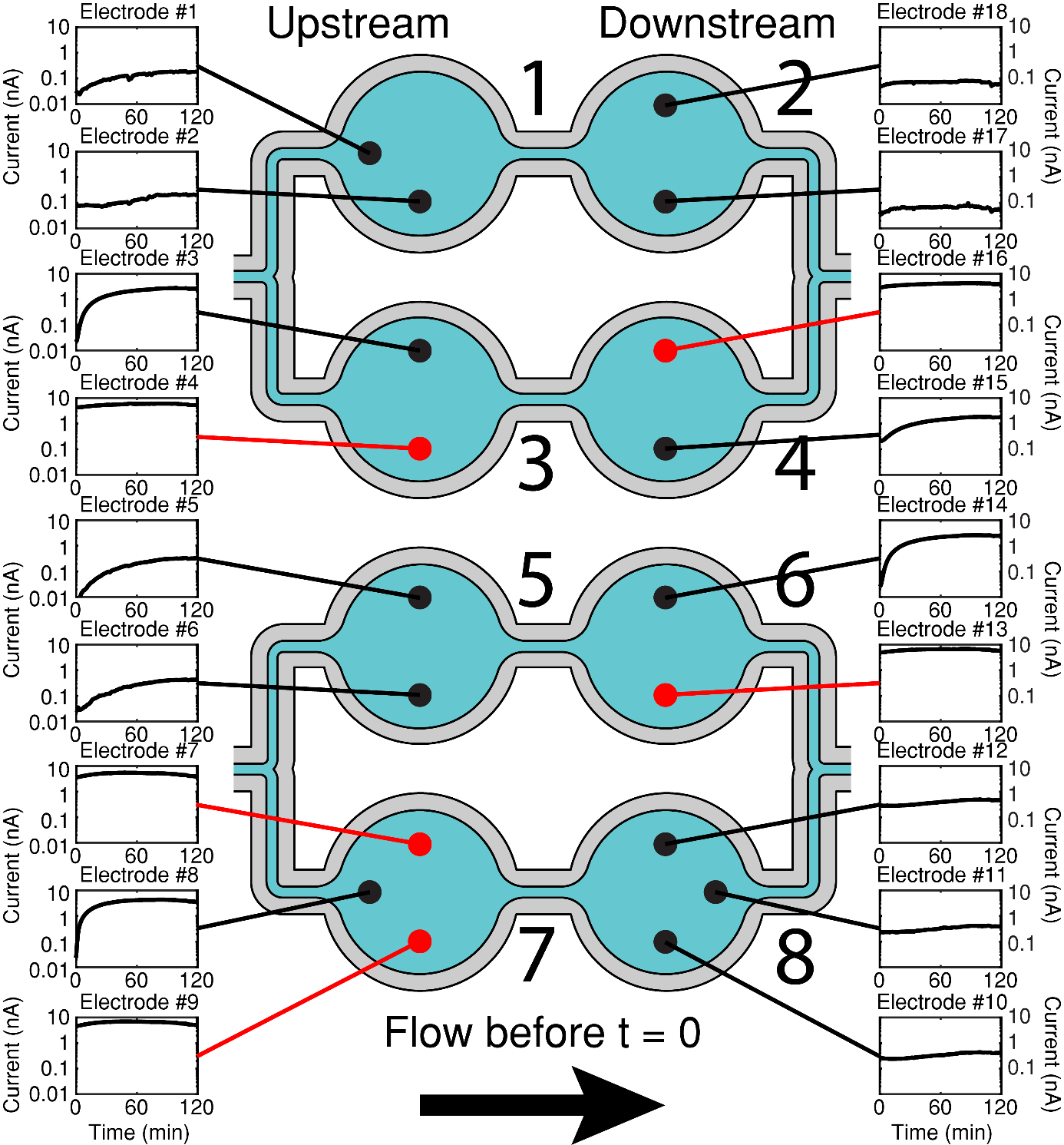


Supplementary Figure 3: **Diffusion processes within our hanging-drop-integrated enzymatic biosensor system.** The working electrodes feature a diameter of 0.4 mm, therefore, currents of 1 nA correspond to a current density of 2 nA mm^-2^. Main figure shows the schematic of the microfluidic network with the numbering of the hanging-drop compartments from 1 to 8. Upstream electrodes are odd-numbered, and downstream electrodes are even-numbered. The special functionalization of electrodes is shown: red-colored electrodes are GOx-functionalized and black electrodes are bare-hydrogel-coated. Flow was applied before the 0-minute timepoint. At 0 minutes, the flow was stopped, and H_2_O_2_ could accumulate and diffuse within the system. The measured current (nA) is shown for all 18 electrodes within the system over the entirety of the experimental measuring time of two hours. The target hanging-drop height prior to the start of the measurement was 800 μm.


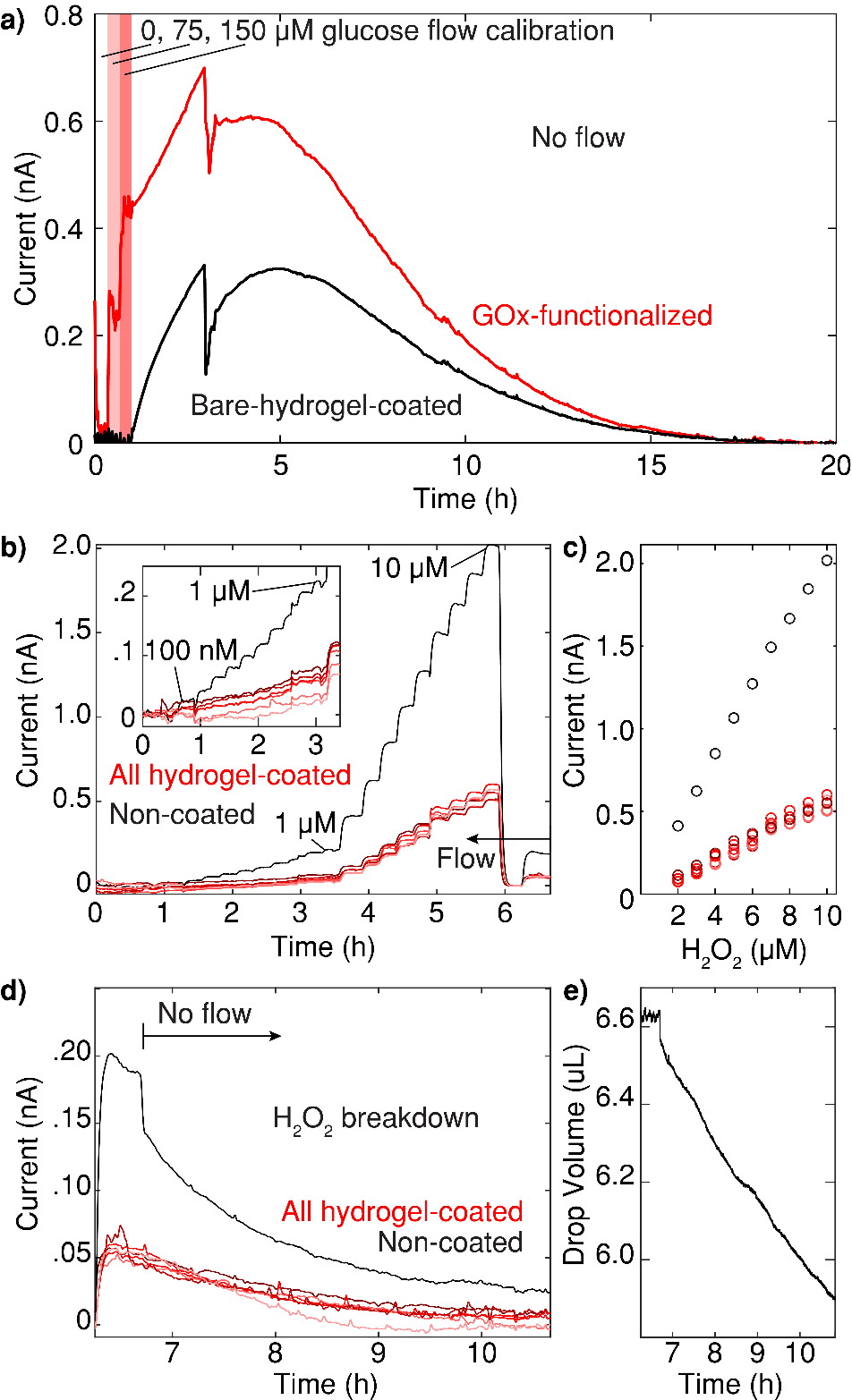


Supplementary Figure 4: **Hydrogen peroxide consumption in the hanging-drop compartments.** The working electrodes feature a diameter of 0.4 mm, therefore, currents of 1 nA correspond to a current density of 2 nA mm^-2^. a) Initial 3-point calibration and measurement in a drop containing no spheroid. The experiment was interrupted abruptly after a 2-hour measurement period (sharp current transient at 3 h), and the current was measured over the course of 20 hours. This long-term measurement shows complete exhaustion of available glucose, as it is enzymatically converted to yield H_2_O_2_ (initial current increase), followed by H_2_O_2_ oxidation by a GOx-functionalized electrode and by a bare-hydrogel-coated electrode during a measurement without flow. b) Flow-calibration of H_2_O_2_ featuring a single, non-coated blank platinum electrode (black line) and several GOx-functionalized and bare-hydrogel-coated electrodes (shades of red). In a first step, H_2_O_2_ is ramped up from 100 nM to 1 μM in 100 nM increments (figure inset), resulting in currents up to 0.21 nA. Afterward, H_2_O_2_ is ramped up from 2 μM to 10 μM in 1 μM increments, resulting in currents up to 2.1 nA. c) The resulting H_2_O_2_ calibration curve is shown for the higher-concentration ramp up for clarity. The calibration shows linearity over the entire range and demonstrates that all coatings have the same effect on H_2_O_2_ diffusion regardless of their composition. d) After the calibration in subfigure b), a buffer with no H_2_O_2_ is injected, followed by a buffer with 1 μM H_2_O_2_. Immediately after the 1 μM H_2_O_2_ buffer is injected, flow is stopped to allow H_2_O_2_ breakdown for over 4 hours. e) Evaporation during that time is shown by monitoring the drop volume over time. This shows that the initial current increase in subfigure a) is only due to enzymatic conversion of glucose, yielding H_2_O_2_, and not a consequence of upconcentration due to evaporation.


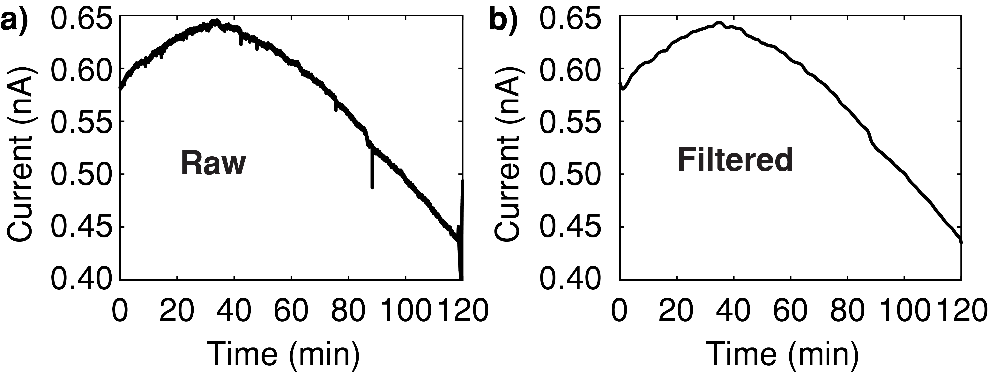


Supplementary Figure 5: **Low-pass filtering effect on raw data.** The working electrodes feature a diameter of 0.4 mm, therefore, currents of 1 nA correspond to a current density of 2 nA mm^-2^. a) Raw current data read from a single electrode during a typical experiment. b) Data filtered using a low-pass filter, described in the manuscript.


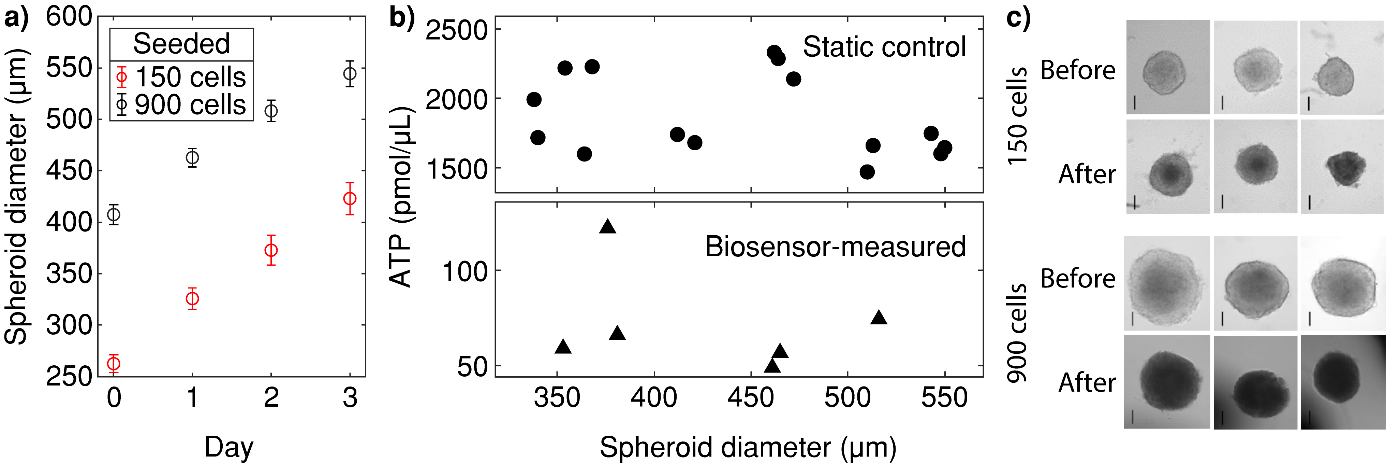


Supplementary Figure 6: **HCT116 spheroid characterization.** a) Spheroid diameters over multiple days in static culture at two seeding densities: 150 or 900 cells. Standard deviation is shown for 30a spheroids imaged in the 96-well culture plate with the plate scanner. Day 0 represents the size of spheroids 4 days after seeding, right before the first metabolism measurement. b) ATP values, normalized by spheroid volume, for cells within spheroids under control conditions (top – equivalent to the “Before” condition in the next subfigure) and after three metabolism measurements within our hanging-drop compartments (bottom – equivalent to the “After” condition in the next subfigure. This data set shows that – even though the glucose concentration has been reduced from 11 mM to 150 μM (a 73-fold decrease), the ATP content has only been reduced approximately 30-fold. c) Image of spheroids before and after a metabolism measurement experiment within the hanging-drop compartments. Scale bar is 100 μm.


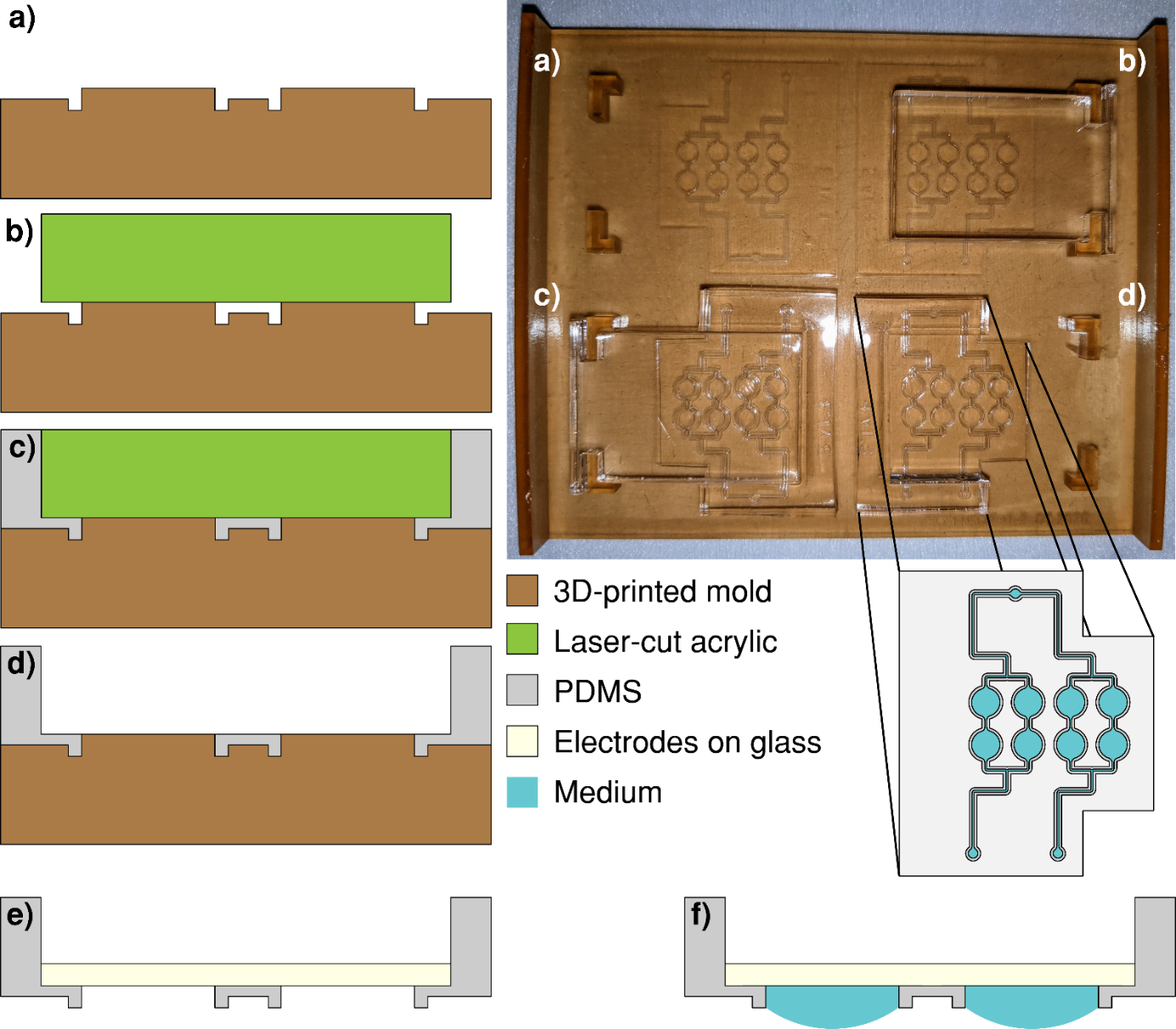


Supplementary Figure 7: **Steps to fabricate the microfluidic channels.** All steps are shown in their cross-section. a) We used a thermally cured 3D-printed mold ordered from MicroLitho UK. The mold is shown in the micrograph at the top right. b) A laser-cut piece of acrylic, custom-fitted for the pins featured on our 3D-printed mold, was put in place, covering the protruding features on the 3D-printed mold. The laser-cut acrylic is also shown in the micrograph at the top right. c) PDMS was poured around the acrylic and on the 3D-printed mold. A slow degassing process allowed the PDMS to infiltrate the 250 μm thin gap between the acrylic and 3D-printed mold and allowed all gas bubbles to escape the microfluidic structures. The PDMS under the laser-cut acrylic is shown in the micrograph at the top right. d) After a 1 hour curing at 80°C, the laser-cut acrylic was removed, and the PDMS was cured for another hour at 80°C. The end result was a 250-μm-thin layer of PDMS with through-holes at each hanging-drop compartment. The PDMS on the 3D-printed mold is shown in the micrograph at the top right. e) The PDMS was then demolded and bonded onto the prepared glass substrate carrying the electrodes via oxygen plasma activation. f) Once the bonding was completed, the electrodes could be functionalized with hydrogel layers and the devices could be filled with the required medium.


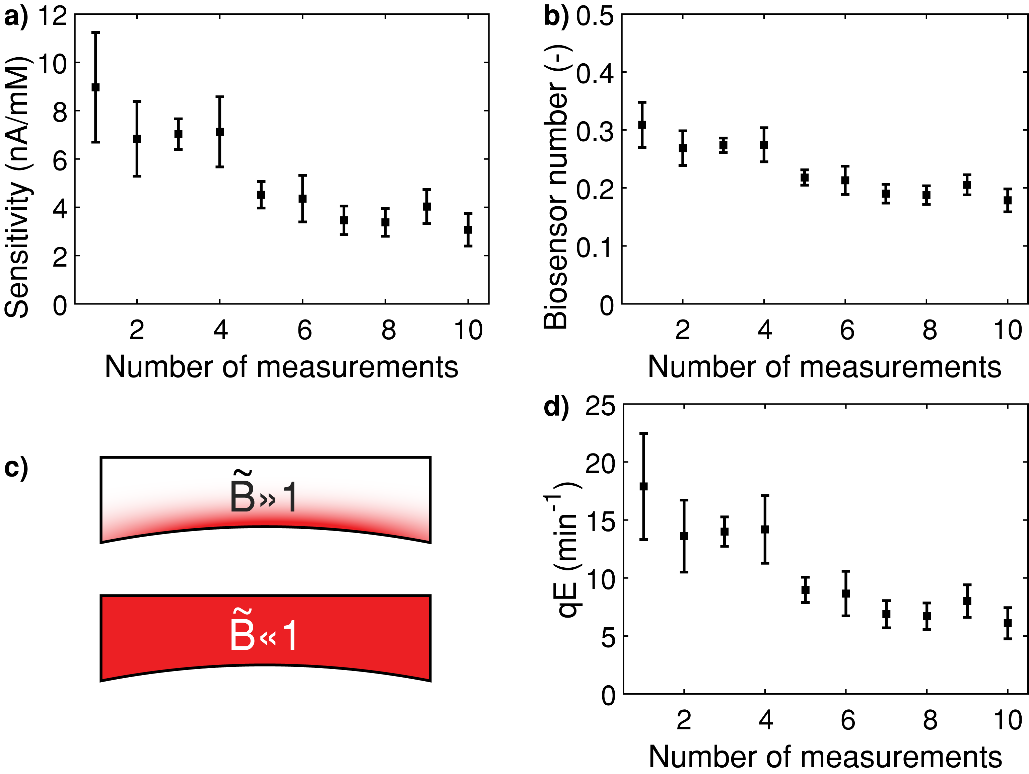


Supplementary Figure 8: **Experimental values of** $\boldsymbol{b}$**,** $\boldsymbol{q}_{\boldsymbol{E}}$ **and** $\tilde{\boldsymbol{B}}$ **for a single chip with a typical functionalization used for 10 measurements over a three-day period.** Standard deviations over the measurements of eight GOx-functionalized electrodes in one chip are shown for each data point. a) Values of the sensitivity $b$ found from the slope of a calibration curve in nA mM^-1^. The sensitivity decreased over time due to a deactivation of the glucose oxidase enzyme by excessive exposure to H_2_O_2_. The largest sensitivity drop happened on the first day due to running a linear-range calibration measurement for sensor characterization. b) Using equation (6), the value of the biosensor number $\tilde{B}$ is found from the sensitivity and plotted. c) A low $\tilde{B}$ implies enzyme activity throughout the hydrogel, whereas a large $\tilde{B}$ implies enzyme activity only at the hydrogel-medium interface. In our case, $\tilde{B}$ was lower than 1. This implies a heterogeneous activity of enzyme in the hydrogel. d) Finally, knowing the value of $\tilde{B}$, we can extract the experimental value of $q_{E}$ with equation (7). We plot typical values of $q_{E}$ in min^-1^ for the previous experimental sensitivities.


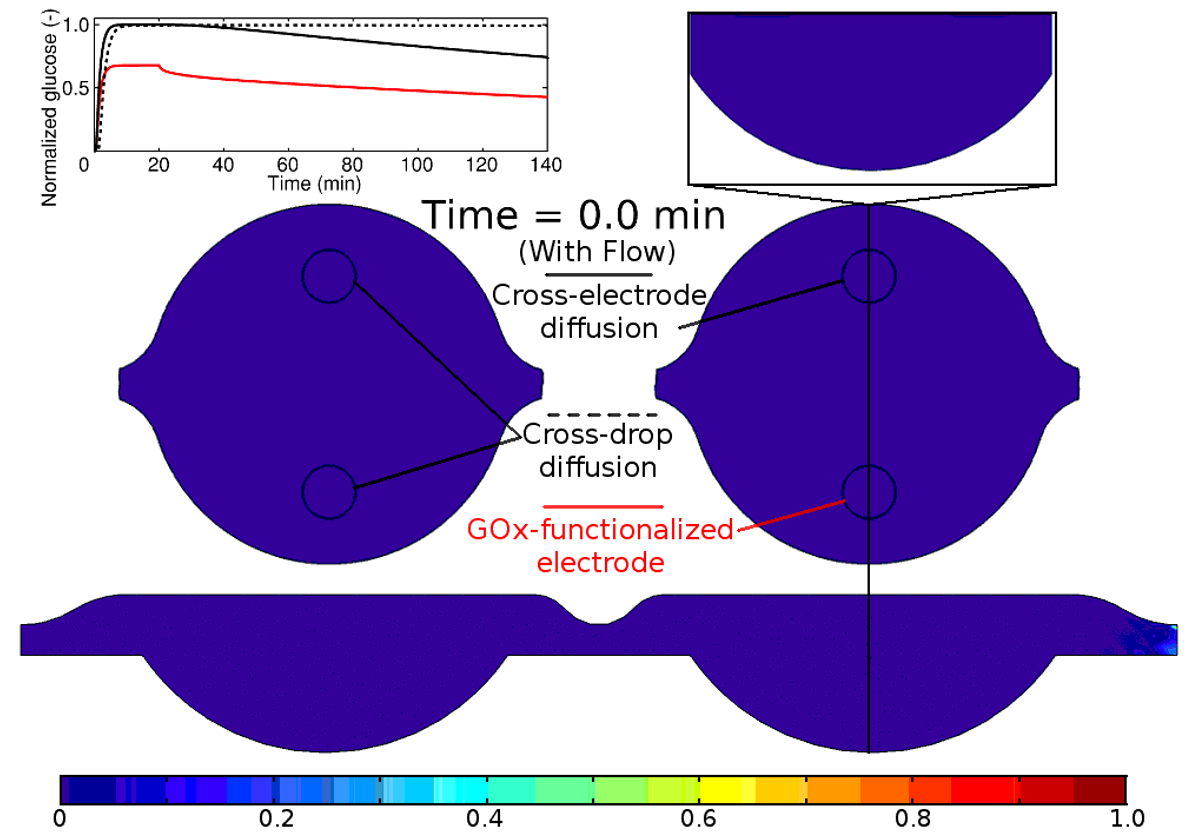


Supplementary Movie 1: Video showing the prescription of 150 μM of glucose within our chip. The result is normalized by this prescribed concentration. A 20-minute flow prescription (only 7.5 minutes are shown) from right to left within a hanging-drop compartment with an 800-μm-high hanging drop. This prescription phase is followed by 120 minutes of stopped flow, during which the GOx-functionalized electrode can oxidize glucose, yielding H_2_O_2_. This shows the dynamics of Figure S1a.


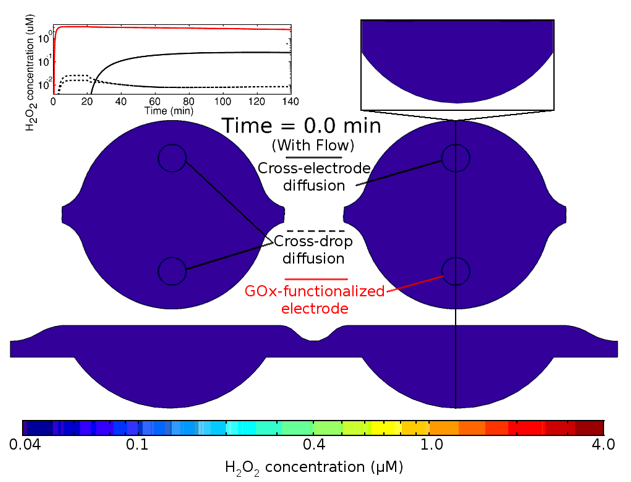


Supplementary Movie 2: Video showing the production of H_2_O_2_ caused by the prescription of 150 μM of glucose within our chip containing a single upstream GOx-functionalized electrode. The concentration is shown with a logarithmic scale to emphasize relative concentration values from electrode to electrode. We apply a 20-minute flow prescription (only 7.5 minutes are shown) from right to left within a hanging-drop compartment with an 800-μm-high hanging drop, which shows downstream H_2_O_2_ transport. This prescription phase is followed by 120 minutes of stopped flow, during which the GOx-functionalized electrode can oxidize glucose, yielding H_2_O_2_. The electrodes are also seen oxidizing H_2_O_2_ for the amperometric readout. This movie shows the dynamics of Figure S1c.
