# Supplementary figures and images for: Modeling and measuring glucose diffusion and consumption by colorectal cancer spheroids in hanging drops using integrated biosensors
Source: Microsyst Nanoeng. 2022 Feb 1;8:14. doi: 10.1038/s41378-021-00348-w (PMC8803859; doi:10.1038/s41378-021-00348-w)

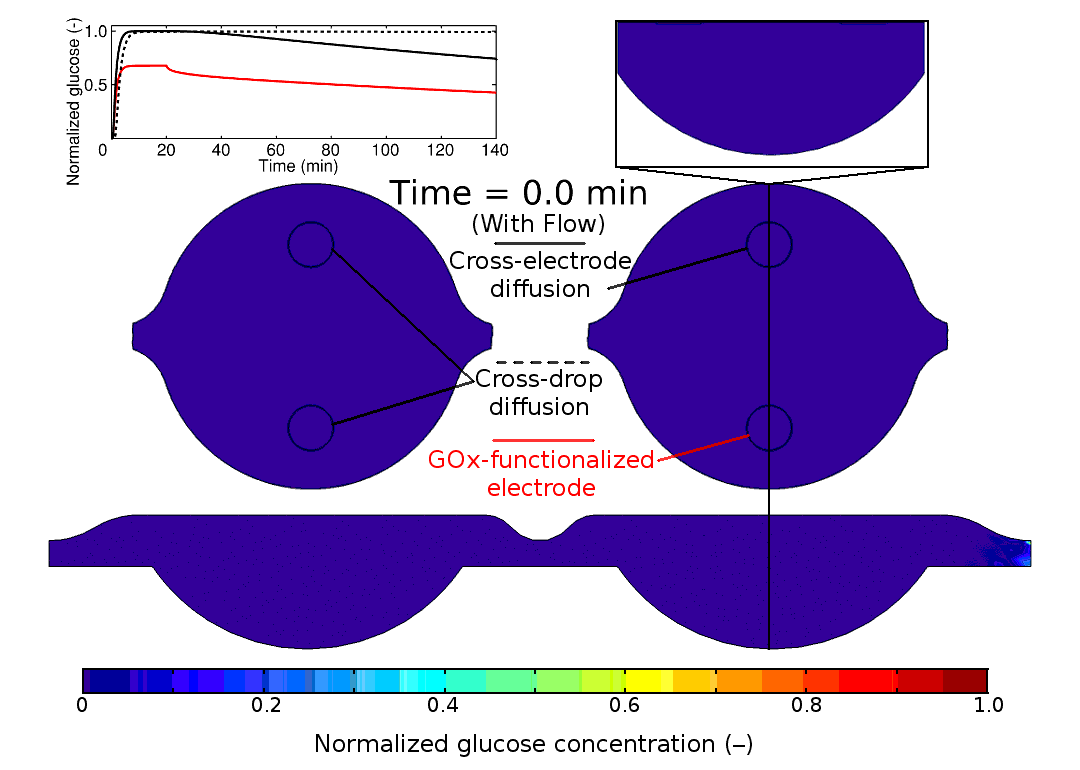

Supplement: Supplementary file 2 — Supplementary Movie 1 [file 41378_2021_348_MOESM2_ESM.gif]

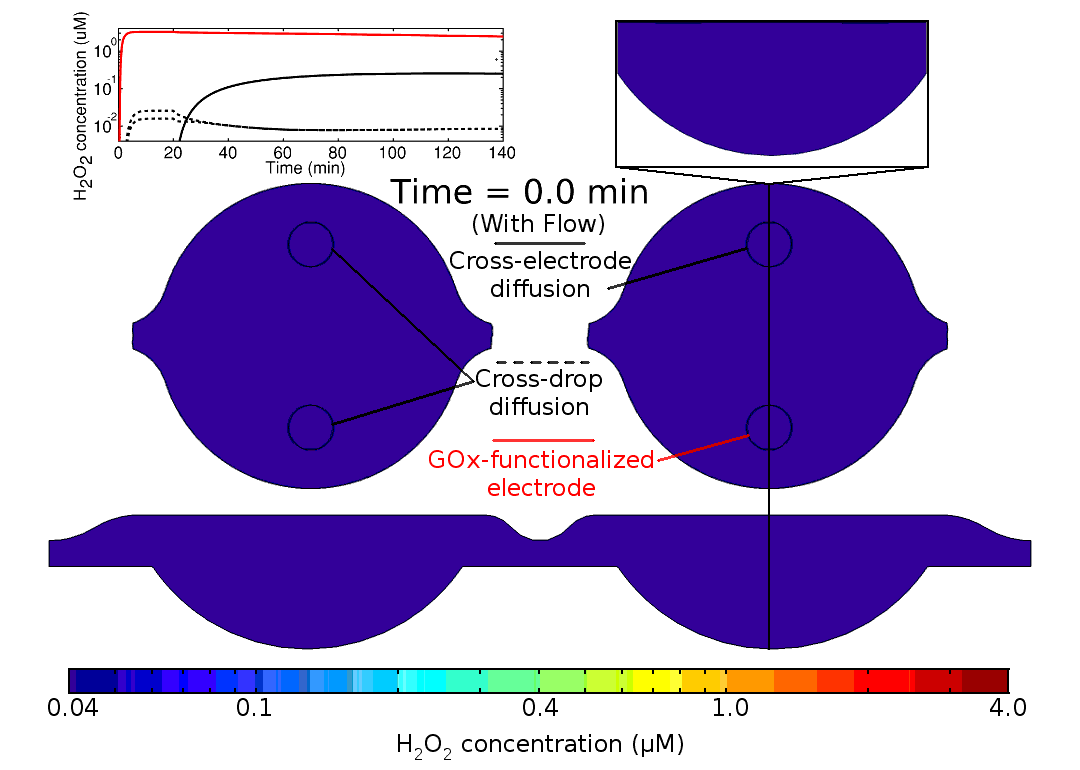

Supplement: Supplementary file 3 — Supplementary Movie 2 [file 41378_2021_348_MOESM3_ESM.gif]
